# Prophylactic Use of Tranexamic Acid to Prevent Postpartum Hemorrhage in High-Risk Cesarean Deliveries: A Systematic Review and Meta-Analysis

**DOI:** 10.3390/jcm15124630

**Published:** 2026-06-15

**Authors:** Xochitl Sandoval López, Hazel C. García, Cesar M. Gavidia, Karina V. Alam, Zaida I. Álvarez, Mirna E. Meléndez, David A. Tejada

**Affiliations:** 1 National Institute of Health, San Salvador 1101, El Salvador; xochitlsandoval2005@gmail.com (X.S.L.); hazel.garcia@salud.gob.sv (H.C.G.); cesar.gavidia@salud.gob.sv (C.M.G.); karina.alam@salud.gob.sv (K.V.A.); zaida.alvarez@salud.gob.sv (Z.I.Á.); elenamega93@gmail.com (M.E.M.); 2 Governance Unit, National Institute of Health, San Salvador 1101, El Salvador; 3 National Health Observatory, National Institute of Health, San Salvador 1101, El Salvador

**Keywords:** postpartum hemorrhage, tranexamic acid, cesarean section, high-risk pregnancy

## Abstract

**Background/Objective**: Postpartum hemorrhage is a leading cause of maternal morbidity and mortality, particularly among high-risk women undergoing cesarean section. This systematic review and meta-analysis evaluated the efficacy and safety of prophylactic tranexamic acid in high-risk cesarean sections. **Methods**: A systematic review and meta-analysis of randomized controlled trials was conducted. Risk of bias was assessed using RoB 2.0 and certainty of evidence was evaluated with GRADE. Random-effects meta-analyses, subgroup analyses and sensitivity analyses were performed. PROSPERO: CRD420251087054. **Results**: Nine randomized controlled trials involving 1776 participants were included. Tranexamic acid reduced total blood loss (MD −300.78; 95% CI −459.78 to −157.77), with greater efficacy when administered 15–20 min before incision (SMD −0.61; 95% CI −0.82 to −0.39). It also reduced intraoperative blood loss (MD −256.71 mL; 95% CI −375.04 to −138.39), blood loss >1000 mL (RR 0.24; 95% CI 0.14 to 0.41), additional uterotonics (RR 0.37; 95% CI 0.24 to 0.58), blood transfusions (RR 0.30; 95% CI 0.22 to 0.40), and complementary surgical interventions (RR 0.35; 95% CI 0.16 to 0.78). **Conclusions**: Prophylactic tranexamic acid may reduce blood loss in high-risk cesarean deliveries, particularly when administered 15–20 min before skin incision. It may decrease total and intraoperative blood loss and blood loss exceeding 1000 mL. It also likely reduces the postoperative decline in hemoglobin, the need for additional uterotonics, blood transfusions, and complementary surgical interventions; however, its effect on hematocrit remains uncertain.

## 1. Introduction

Postpartum hemorrhage (PPH) is one of the leading causes of maternal morbidity and mortality worldwide and is particularly relevant in women who have undergone cesarean section (C-section) [1]. This type of hemorrhage is defined as blood loss exceeding 1000 mL after delivery or accompanied by symptoms or signs of hypovolemia within 24 h after birth [2], which can lead to serious complications or death [3]. Despite advances in obstetric care, PPH remains a major challenge, with direct implications for maternal health and healthcare costs [4]. Globally, the proportion of cesarean deliveries is increasing [5], accounting for approximately 30% of deliveries in developed countries and 20% of all deliveries worldwide [6]. It is estimated that between 1% and 5% of these procedures may be complicated by PPH [7]. Maternal mortality attributed to PPH remains high in some regions [8], with the prevalence varying by region, ranging from 2.4% to 12.1% [9].

Women considered at high risk are more likely to suffer from PPH due to factors such as a history of hemorrhage, multiple pregnancies, placenta previa, or placental abruption, among others. These conditions increase the complexity of obstetric management and highlight the need for effective prophylactic interventions to reduce the incidence and severity of hemorrhage in this vulnerable group.

In this context, tranexamic acid (TXA) has been proposed to reduce bleeding in women undergoing C-section [10]. TXA is an antifibrinolytic agent that inhibits fibrinolysis and helps stabilize clot formation [11]. Its use has become widespread in various surgical and trauma settings, and it has also been evaluated as a prophylactic intervention for PPH [9]. However, evidence of its efficacy compared with placebo or standard treatment remains under investigation [12].

Therefore, this systematic review aimed to evaluate the efficacy and safety of prophylactic TXA for preventing PPH in women undergoing high-risk C-section, compared with placebo or standard treatment.

## 2. Methods

### 2.1. Study Design

A systematic review with meta-analysis was conducted following the recommendations of the Preferred Reporting Items for Systematic reviews and Meta-Analyses (PRISMA) 2020 statement [13]. The completed PRISMA 2020 checklist is provided in the Supplementary Material as Table S4. The protocol for this systematic review and meta-analysis was registered in the PROSPERO database under the registration number CRD420251087054. The record was published on 4 July 2025, and is available at: https://www.crd.york.ac.uk/PROSPERO/view/CRD420251087054 (accessed on 5 May 2026).

### 2.2. Searches

A systematic search was conducted in PubMed, Scopus, Web of Science, EMBASE, CINAHL, Cochrane Library, ClinicalTrials.gov, and International Clinical Trials Registry Platform of the World Health Organization databases from their inception to 3 July 2025. For each database, a tailored search strategy was applied, using free-text terms in all cases, the Medical Subject Headings thesaurus in PubMed, and the Emtree thesaurus in EMBASE and Scopus. The main search terms included “postpartum hemorrhage”, “tranexamic acid”, “cesarean section”, and “High-Risk Pregnancy”. No language or publication date restrictions were applied. Additionally, all reference lists from relevant studies and included review articles were manually searched for other potentially eligible trials (Table S1).

### 2.3. Eligibility Criteria

All randomized controlled trials that evaluated pregnant women undergoing cesarean delivery and compared prophylactic TXA in a dose of 1 g with placebo or standard treatment were included. The studies included women aged 18 years or older with medical or obstetric conditions associated with a high risk of postpartum hemorrhage. High risk was defined according to the criteria used in the primary studies and required the presence of at least one recognized risk factor. These included a history of postpartum hemorrhage, placental abnormalities, placental abruption, multiple gestation, polyhydramnios, fetal macrosomia, previous cesarean delivery, obesity, hypertensive disorders of pregnancy, uterine fibroids, prolonged labor, and maternal anemia. Studies involving women with hypersensitivity to TXA, hematological disorders with a predisposition to bleeding, a history of thromboembolism, or ongoing anticoagulant treatment were excluded.

### 2.4. Outcomes

The primary outcomes were total blood loss, blood loss within two hours postpartum, and the incidence of postpartum hemorrhage. Total blood loss was defined as cumulative blood loss measured or estimated within 48 h after cesarean delivery, including formula-based estimates derived from perioperative hemoglobin or hematocrit values reported within the same period. Blood loss within two hours postpartum corresponded to the volume lost during delivery and the first two hours after delivery. Postpartum hemorrhage was defined as blood loss greater than 1000 mL or the need for red blood cell transfusion within two days after delivery.

Secondary outcomes include changes in hemoglobin and hematocrit. The occurrence of mild adverse events, such as diarrhea, nausea, vomiting and headache, as well as serious adverse events associated with the procedure, such as thromboembolic complications, hypersensitivity reactions and other medical complications, was systematically documented.

The length of hospital stay was also evaluated to estimate the impact of the intervention on recovery time, the proportion of patients requiring blood transfusion, additional surgical interventions such as vascular ligation, uterine compression sutures, or hysterectomy and the need for additional administration of uterotonics.

### 2.5. Selection Process

The records obtained from the electronic searches were exported to EndNote, where they were consolidated into a library, and duplicates were removed. The library was then uploaded to the Rayyan web platform for initial screening by title and abstract. Studies selected in the initial screening were evaluated in full text for a new review process. Eligible studies were included in the review, and the data extraction process was performed. This stage was independently evaluated by reviewers XSL, DAT, HCG, CMG, KVA, and ZIA. Conflicts were resolved at each stage by team consensus.

### 2.6. Data Extraction

Four reviewers extracted data from each study independently, using a predesigned Microsoft Excel spreadsheet. For each analysis, we collected information on the author, year of publication, country, study design, number of participants per intervention group, mean age per group, eligibility criteria, intervention and comparator characteristics, and primary and secondary outcomes.

When multiple publications appeared to report data from the same trial population, they were treated as companion reports of a single study. Outcome data were extracted only once for each meta-analysis to avoid participant duplication. When overlapping outcome data were available, the most complete or detailed report was prioritized, and the companion publication was used to verify study characteristics and outcome consistency.

### 2.7. Assessment of Risk of Bias

The risk of bias was assessed via the RoB 2.0 tool by the reviewers DAT, HCG, CMG, KVA, and ZIA. RoB was classified by domain as low, with some concerns, or high. Disagreements were resolved through discussion with a sixth reviewer (XSL) [14].

### 2.8. Data Synthesis

The meta-analysis was performed via a random effects model, and the inverse variance method was used to estimate the effects of TXA compared with those of the placebo. The variance between studies (τ^2^) was estimated via the Paule-Mandel method.

For continuous blood loss outcomes, raw mean differences (MDs) in milliliters with 95% confidence intervals (95% CIs) were used as the primary effect measure to improve clinical interpretability. Given the variability in blood loss measurement methods, study populations, and clinical settings across the included studies, standardized mean differences (SMDs) were also calculated as sensitivity analyses to assess the robustness of the findings. These sensitivity analyses are presented in the Supplementary Material.

For dichotomous outcomes, the effect was quantified using the relative risk (RR) with 95% CI. In studies with no events in one or both groups, a continuity correction was applied. The confidence intervals were adjusted via the Hartung-Knapp-Sidik-Jonkman method for meta-analyses with more than five studies or the profile likelihood method for those with five or fewer studies.

We assessed heterogeneity among studies using the $I^{2}$ statistic and between-study variance using τ^2^. $I^{2}$ values below 30%, between 30% and 60%, and above 60% were interpreted as low, moderate, and high heterogeneity, respectively. Heterogeneity was explored through subgroup analyses according to timing of TXA administration, method of blood loss quantification, sample size, geographic region, type of cesarean delivery, and inclusion of placenta previa in the high-risk criteria, when information was available. Sensitivity analyses, leave-one-out analyses, and formal influence analyses were also performed. Meta-regression was considered but was not performed because of the limited number of studies per outcome.

Publication bias and small-study effects were not formally assessed using funnel plots or Egger’s test because fewer than 10 studies were available per outcome, which limited the reliability and interpretability of these methods.

Statistical analyses were performed in RStudio version 4.4.2. Meta-analyses of dichotomous and continuous outcomes were conducted using the meta package, with the metabin and metacont functions, respectively. Formal influence analyses were performed using the metafor package.

### 2.9. GRADE Assessment

The certainty of the evidence and the level of recommendation were assessed via the GRADE methodology, which considers the domains of risk of bias, inconsistency, indirect evidence, imprecision, and publication bias. The assessment was performed for each outcome and summarized in summary of evidence (SoE) tables, developed via the online software GRADEpro GDT, available at: https://www.gradepro.org (accessed on 1 May 2026) [15].

## 3. Results

**Figure 1:**
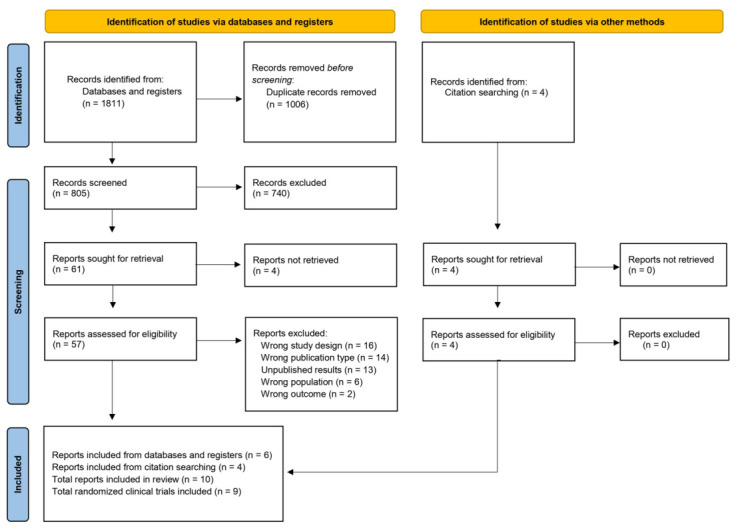
PRISMA flow diagram [13].

A total of 1811 records were identified through database and trial registry searches. After removing 1006 duplicate records, 805 records remained for title and abstract screening, of which 740 were excluded for not meeting the inclusion criteria. Sixty-one full-text reports were sought for retrieval, but four were not available. Therefore, 57 reports were assessed for eligibility, of which 51 were excluded because of wrong study design (*n* = 16), wrong publication type (*n* = 14), unpublished results (*n* = 13), wrong population (*n* = 6), or wrong outcome (*n* = 2). Ultimately, ten reports corresponding to nine randomized clinical trials were included in the review [16,17,18,19,20,21,22,23,24,25], including four reports [16,20,22,23] identified through manual searching of reference lists (Figure 1).

Among the included records, Abdel-Fatah et al., 2021 [16], and Abdel-Fatah et al., 2022 [17] were identified as companion publications arising from the same randomized controlled trial, as they shared the same author group, institution, recruitment period, sample size, intervention, comparator, and main outcome estimates. Although neither publication cited the other, both reports were considered to originate from a single trial. The 2021 report provided the more detailed outcome information, including blood loss during cesarean section and postoperative hemoglobin and hematocrit values, whereas the 2022 report was used to verify study characteristics and duplicated outcome data. Outcome data were extracted only once for each meta-analysis to avoid participant duplication. For overall study counts, these two publications were therefore counted as a single study (Table S5).

### 3.1. Characteristics of Participants and Included Studies

Nine randomized clinical trials, reported in ten articles and published between 2019 and 2025, were included. The studies were conducted in Asia and Africa and comprised 1776 women, with sample sizes ranging from 50 to 800 participants. In general, reported baseline characteristics were comparable between the intervention and control groups within each trial. Maternal age and the hemorrhage-related clinical factors reported in each study did not show significant between-group differences (*p* > 0.05). However, the criteria used to define high-risk status varied across trials and included conditions such as placenta previa, anemia, previous cesarean delivery, hypertensive disorders of pregnancy, multiple gestation, fetal macrosomia, polyhydramnios, and previous postpartum hemorrhage. Cesarean delivery type also varied across studies, with some trials including elective procedures and others including emergency cesarean deliveries or not clearly reporting this distinction. This variability was considered when interpreting clinical heterogeneity and generalizability.

Blood loss was assessed using gravimetric methods, based on the weighing of surgical drapes and towels, as well as formula-based approaches, including estimated blood loss (EBL) and the Nadler formula. Follow-up ranged from 24 to 48 h in most studies, although some trials extended follow-up to four or six weeks (Table 1).

### 3.2. Risk of Bias Assessment

**Figure 2:**
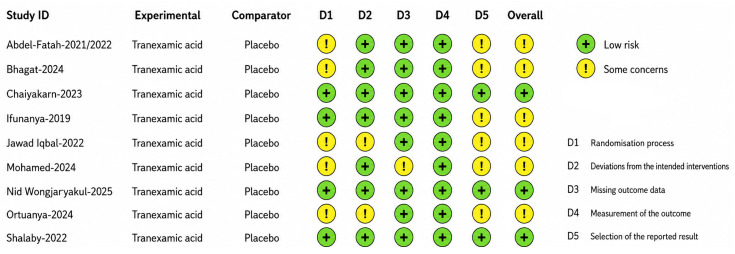
Risk of bias in included studies [16,17,18,19,20,21,22,23,24,25].

Overall, six studies were judged as having some concerns regarding risk of bias and three as having low risk of bias. The main domains contributing to these judgments were bias arising from the randomization process in four studies, bias due to deviations from intended interventions in two studies, and bias in selection of the reported result in six studies. No major concerns were identified for bias due to missing outcome data or bias in measurement of the outcome (Figure 2).

### 3.3. Primary Outcomes

#### 3.3.1. Total Blood Loss

**Figure 3:**
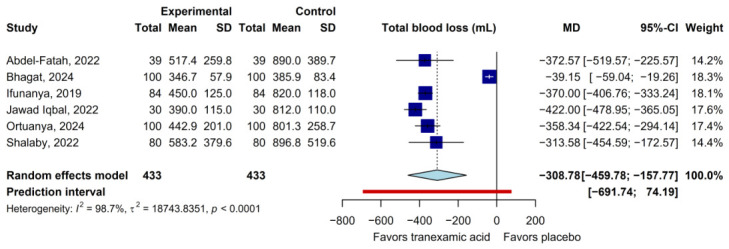
Meta-analysis of the efficacy of tranexamic acid compared with placebo or standard care in reducing total blood loss [17,18,20,21,24,25].

The pooled analysis of six studies, including 866 participants, suggests that prophylactic TXA results in a substantial reduction in total blood loss in high-risk pregnant women undergoing C-section (MD −300.78; 95% CI −459.78 to −157.77). Considerable heterogeneity was observed across studies ($I^{2}$ 98.7%; *p* < 0.0001), indicating substantial variability in effect estimates. The certainty of the evidence (CoE) was moderate (Figure 3).

#### 3.3.2. Sensitivity and Subgroup Analyses

Given the considerable heterogeneity observed in the overall analysis of total blood loss, sensitivity and subgroup analyses were conducted to explore the robustness of the findings and potential clinical or methodological sources of between-study variability.

In the sensitivity analyses, exclusion of Bhagat et al., 2024 [18] substantially changed the heterogeneity estimates. After removing this study, the pooled effect remained in favor of TXA, and no heterogeneity was observed (MD −377.56 mL; 95% CI −404.40 to −350.71; $I^{2}$ 0%; τ^2^ 0; *p* 0.4567) (Figure S1). This finding suggests that Bhagat et al., 2024 [18] had an important statistical influence on the inconsistency observed in the overall analysis; however, this result should be interpreted as exploratory given the limited number of included studies.

Subgroup analyses showed a pattern consistent with these findings. According to the method used to quantify blood loss, studies using the Nadler formula showed a substantial reduction in total blood loss, with moderate and non-significant heterogeneity (MD −391.27 mL; 95% CI −441.37 to −341.16; $I^{2}$ 55.8%; *p* 0.1327). Studies using the EBL formula also showed reduced blood loss, with no observed heterogeneity (MD −350.65 mL; 95% CI −409.09 to −292.22; $I^{2}$ 0%; *p* 0.5713). In contrast, Bhagat et al., 2024, classified under the gravimetric method, showed a smaller effect estimate (MD −39.15 mL; 95% CI −59.04 to −19.26), which may partly explain the variability observed across studies (Figure S2).

A similar pattern was observed in other exploratory subgroup analyses, including placenta previa status, geographic region, sample size, duration of follow-up, type of cesarean delivery, and mean maternal age. Overall, effect estimates favored TXA in most evaluated strata; however, the precision of the estimates and the magnitude of heterogeneity varied across subgroups (Figures S3–S7). In addition, analyses using standardized mean differences showed a consistent direction of effect in favor of the intervention (Figures S8–S16).

Regarding timing of administration, the standardized mean difference analysis was retained. In studies in which TXA was administered within 10 min before skin incision, the reduction in total blood loss was larger, although substantial residual heterogeneity was observed (SMD −2.31; 95% CI −3.50 to −1.13; 4 studies; 506 participants; low certainty of evidence; $I^{2}$ 94.7%). In studies in which TXA was administered 15–20 min before cesarean delivery, the effect estimate was smaller but more consistent (SMD −0.61; 95% CI −0.82 to −0.39; 2 studies; 360 participants; moderate certainty of evidence; $I^{2}$ 0%; *p* 0.51) (Figure S8).

Overall, these subgroup analyses should be interpreted as exploratory because several strata included few studies and no statistically significant differences between subgroups were identified. Therefore, these findings should not be considered conclusive evidence of effect modification.

#### 3.3.3. Intraoperative Blood Loss

**Figure 4:**
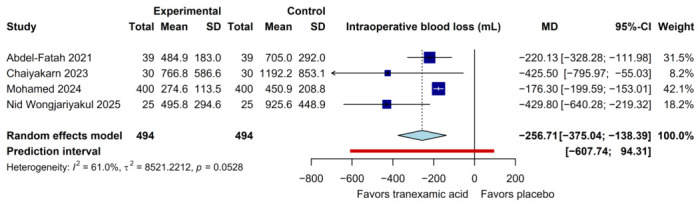
Meta-analysis of tranexamic acid versus placebo or standard care for reducing intraoperative blood loss [16,19,22,23].

Furthermore, in the pooled analysis of four studies including 988 participants, TXA was associated with lower intraoperative blood loss compared with control (MD −256.71 mL; 95% CI −375.04 to −138.39). Moderate between-study heterogeneity was observed ($I^{2}$ 61.0%; *p* 0.0528), and the certainty of evidence for this outcome was moderate (Figure 4).

#### 3.3.4. Sensitivity Analysis for Intraoperative Blood Loss

A sensitivity analysis excluding Mohamed et al., was conducted because of limitations in methodological reporting. After exclusion, TXA remained associated with a significant reduction in intraoperative blood loss compared with control (MD −308.91 mL; 95% CI −451.73 to −166.08; $I^{2}$ 46.0%; *p* 0.157). The decrease in heterogeneity suggests that this study contributed partly to between-study variability; however, the direction and statistical significance of the effect remained unchanged, supporting the robustness of the primary finding (Figure S17).

#### 3.3.5. Blood Loss Within 2 h Postpartum

Regarding postpartum blood loss within 2 h, two studies involving 1000 participants evaluated blood loss during the first 2 h after delivery. The pooled analysis showed no statistically significant reduction with TXA compared with placebo or standard care (MD −21.21 mL; 95% CI −53.15 to 10.74), with substantial heterogeneity ($I^{2}$ 90.4%; *p* 0.0013). The CoE for this outcome was very low (Figure S18).

#### 3.3.6. Postpartum Hemorrhage

**Figure 5:**
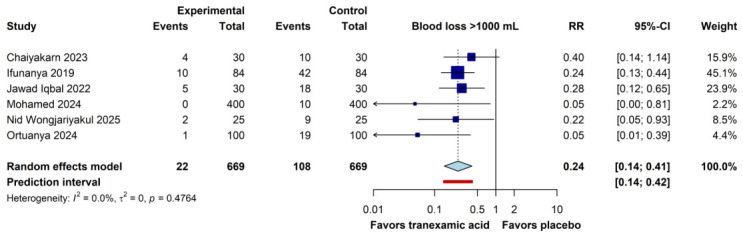
Meta-analysis of tranexamic acid versus placebo or standard care for reducing postpartum hemorrhage greater than 1000 mL [19,20,21,22,23,24].

Finally, six studies including 1338 high-risk women undergoing cesarean section showed that prophylactic TXA likely results in a large reduction in the risk of postpartum hemorrhage greater than 1000 mL compared with control (RR 0.24; 95% CI, 0.14–0.41), with no observed heterogeneity ($I^{2}$ 0.0%; *p* 0.47) and moderate certainty of evidence (Figure 5).

### 3.4. Secondary Outcomes

**Figure 6:**
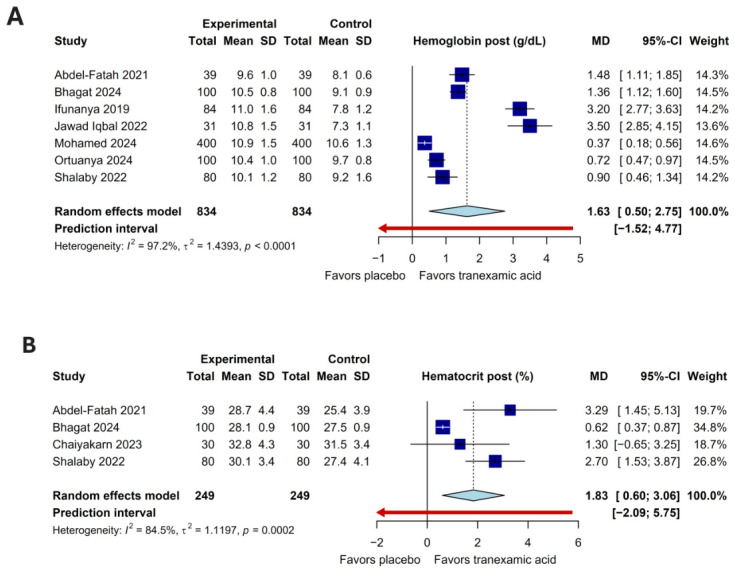
Meta-analysis of postoperative hemoglobin (**A**) and hematocrit [16,18,20,21,22,24,25] (**B**) levels following prophylactic tranexamic acid versus placebo or standard care [16,18,19,25].

TXA was associated with higher postoperative hemoglobin values compared with placebo or standard care. In the pooled analysis of seven studies including 1668 participants, TXA increased postoperative hemoglobin by an average of 1.63 g/dL (MD 1.63 g/dL; 95% CI 0.50 to 2.75), with moderate CoE. Substantial heterogeneity was observed across studies ($I^{2}$ 97.2%; *p* < 0.0001), and the prediction interval was wide and crossed the null value (−1.52 to 4.77), indicating variability in the expected effect across future settings (Figure 6A).

Influence diagnostics suggested that heterogeneity was not driven by a single study. The Baujat plot identified the studies by Jawad Iqbal 2022 and Ifunanya 2019 as having the greatest influence on the pooled effect, whereas Mohamed 2024 contributed to heterogeneity from the opposite direction by reporting the smallest effect estimate (Figure S19). In leave-one-out analyses, $I^{2}$ remained very high after excluding each study individually, ranging from 95.7% to 97.7% (Figure S20). Overall, the heterogeneity appeared to reflect differences in the magnitude of effect across studies rather than inconsistency in the direction of effect, as all studies favored TXA.

Similarly, TXA was associated with higher postoperative hematocrit values compared with placebo or standard care. The pooled analysis of four studies, including 498 participants, showed a mean increase of 1.83 percentage points in postoperative hematocrit among women receiving TXA (MD 1.83%; 95% CI 0.60 to 3.06), with moderate CoE. Substantial heterogeneity was observed across studies ($I^{2}$ 84.5%; *p* 0.0002), and the prediction interval was wide and crossed the null value (−2.09 to 5.75), indicating variability in the expected effect across future settings (Figure 6B). In contrast, TXA showed little to no effect on hospital stay in the pooled analysis of three studies including 910 participants (MD 0.02 days; 95% CI −0.10 to 0.14; $I^{2}$ 23.7%), with very low CoE (Figure S21).

**Figure 7:**
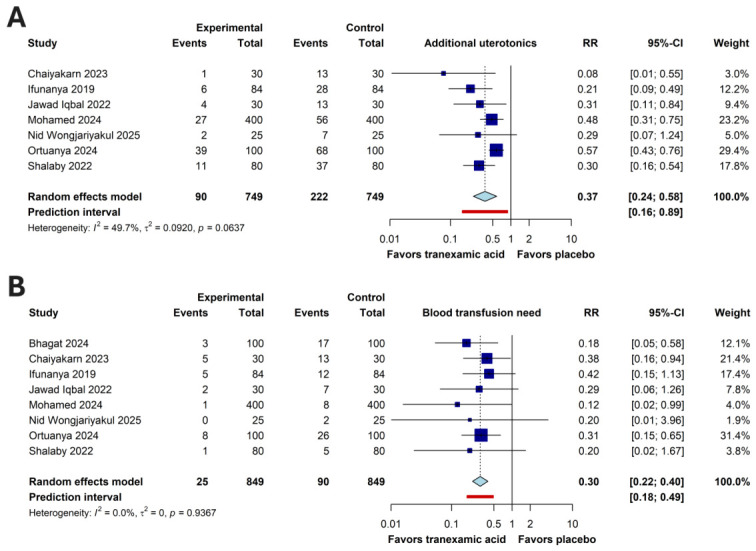
Meta-analysis of additional uterotonic use (**A**) and blood transfusion requirement [19,20,21,22,23,24,25] (**B**) following prophylactic tranexamic acid versus placebo or standard care [18,19,20,21,22,23,24,25].

When evaluating the additional use of uterotonics in women with risk factors, the evidence suggests TXA results in a large reduction in the need for additional uterotonics (RR 0.37; 95% CI 0.24 to 0.58; 7 studies; 1498 participants), with low CoE. Moderate heterogeneity was observed ($I^{2}$ 49.7%; *p* 0.06) (Figure 7A).

The evidence suggests that TXA leads to a large reduction in the need for blood transfusions in women with high-risk pregnancies undergoing C-section (RR 0.30; 95% CI 0.22 to 0.40; 8 studies; 1698 participants), with low CoE (Figure 7B). No heterogeneity was detected ($I^{2}$ 0.0%; *p* 0.9367). Finally, for surgical intervention for postpartum hemorrhage, the evidence suggests TXA results in a large reduction in the need for additional surgical intervention (RR 0.35; 95% CI 0.16 to 0.78; 2 studies; 260 participants), with low CoE. No heterogeneity was observed ($I^{2}$ 0.0%; *p* 0.35) (Figure S22).

### 3.5. Safety Outcomes

Regarding safety outcomes, TXA may have little or no effect on the occurrence of side effects (RR 3.47; 95% CI 0.65 to 18.41; 3 studies; 1050 participants), although the certainty of evidence was very low (Figure S23). Considerable heterogeneity was observed ($I^{2}$ 91.1%; *p* < 0.0001). For serious adverse events, the evidence was also very uncertain (RR 1.00; 95% CI 0.29 to 3.43; 3 studies; 1060 participants), with no detected heterogeneity ($I^{2}$ 0.0%; *p* 1.00) (Figure S24).

## 4. Discussion

The efficacy and safety of TXA versus placebo for the prevention of PPH in high-risk C-sections were evaluated. Prophylactic TXA use has consistently been shown to be associated with a significant reduction in PPH [26]. The high-risk population allowed for the evaluation of TXA effectiveness, demonstrated efficacy in a wide range of high-risk situations, and expanded the current evidence on the prophylactic use of TXA in high-risk C-sections [3,27].

The demonstrated effectiveness suggests that the benefits of TXA persist even in the presence of pathophysiological alterations that could interfere with its mechanism of action [28]. However, the efficacy observed in high-risk populations contrasts with the heterogeneous evidence from other pharmacological strategies to prevent PPH [29,30].

Unlike the WOMAN-2 study, which evaluated TXA as a treatment for established PPH [31], this research focused on its prophylactic use. It acts before the activation of compensatory mechanisms and hyperfibrinolysis, preventing blood loss and stabilizing clots from the early stages. In contrast, its administration once bleeding has already begun inhibits only active fibrinolysis without stopping the initial hemorrhagic cascade [32].

Current recommendations for the management of PPH state that the use of TXA should be considered when initial therapies fail to control bleeding [33], that TXA should be administered within three hours of delivery immediately after bleeding begins [34], or that its use should be part of the initial management of established PPH, alongside uterotonics and mechanical measures [33]. However, the evidence suggests reconsidering these guidelines to include prophylactic use in high-risk populations and to standardize the timing and dosage, as 1 g intravenously is sufficient to inhibit fibrinolysis, whereas 0.5 g does not produce a significant effect [35].

Compared with placebo, TXA was associated with a reduction in total blood loss; however, considerable heterogeneity was observed. Subgroup analyses according to timing of administration suggested a larger effect among studies administering TXA within 10 min before skin incision. Nevertheless, this subgroup showed substantial residual heterogeneity and low certainty of evidence, indicating that the finding should be interpreted cautiously and not as evidence of a purely pharmacological timing effect.

Differences in baseline obstetric risk profiles, cesarean delivery characteristics, blood loss measurement methods, clinical setting, perioperative care protocols, and risk of bias may have contributed to the observed variability. The more consistent estimate observed in the 15–20 min subgroup may be biologically plausible, given the potential for more stable plasma concentrations during the postpartum fibrinolytic activation period [9], which is activated during the peripartum period [27]. However, this interpretation remains exploratory, and further studies are needed to determine the optimal timing of TXA administration and its association with bleeding outcomes.

The effect of TXA on blood loss within the first two hours postpartum remains uncertain. Although previous studies have reported significant reductions during this early period after cesarean delivery [35], the pooled estimate in the present review was not statistically significant and the certainty of evidence was very low. This discrepancy may reflect the small number of contributing studies, substantial heterogeneity, and imprecision. Thus, although TXA reduced total and intraoperative blood loss, its additional benefit during the early postpartum window requires further investigation [9,34,36,37].

The amount of perioperative blood loss also significantly differed in favor of TXA. This effect is probably due to the antifibrinolytic action of TXA during the critical hours of the perioperative period [36]. Similar findings have been reported, showing that the use of a drug is associated with a reduction in the need for transfusions and the risk of severe bleeding [38,39,40,41,42,43].

In relation to blood loss exceeding 1000 mL, TXA was associated with a significant reduction in bleeding, significantly reducing the need for surgical interventions, from conservative techniques such as uterine packing to more invasive procedures such as arterial embolization, hemostatic ligatures, and postpartum hysterectomy [24]. It significantly reduces the need for transfusions, demonstrating its effectiveness in controlling bleeding, supported by better maintenance of hematocrit and hemoglobin postsurgery in favor of the drug [17,24,43,44], reducing the costs associated with transfusions and decreasing the risks inherent in the procedure [45].

These findings are consistent with results reported by other authors, who support its use as a prophylactic intervention to prevent severe PPH, especially in high-risk women [46], and the need for additional interventions such as complementary uterotonics, surgery, and blood transfusions [10,40,41,42,43]. A lower need for complementary uterotonics was observed, and TXA can effectively complement these strategies through its mechanism of action, allowing for a synergistic effect in the management and prevention of PPH [45].

The safety assessment did not show clear differences in the frequency of side effects between TXA and placebo. Reported mild events were generally self-limiting and manageable in the perioperative setting. Similarly, no clear between-group differences were observed for serious adverse events, which is consistent with previous evidence [45,47]. However, current evidence does not demonstrate an increased risk of adverse events, and the available studies remain underpowered to exclude rare thromboembolic complications.

Available evidence suggests that TXA is generally safe during pregnancy and lactation. A meta-analysis of more than 19,000 participants revealed no significant adverse effects in newborns, and drug levels in breast milk accounted for only 1% of maternal serum levels [48]. There were no differences in long-term outcomes, including neurological development, general health, and infant mortality. Occasional complications, such as low birth weight or preterm delivery, are related to maternal conditions rather than the drug. Therefore, breastfeeding is considered safe, and the U.S. Food and Drug Administration classifies TXA as category B during pregnancy [48]. An important limitation of this study is its restricted representativeness, as most included trials were conducted in specific geographic and healthcare contexts, mainly in Asia and Africa. Although baseline characteristics were generally comparable between intervention and control groups within each trial, the criteria used to define high-risk status varied across studies and included heterogeneous obstetric and clinical conditions, such as placenta previa, anemia, previous cesarean delivery, hypertensive disorders of pregnancy, multiple gestation, fetal macrosomia, polyhydramnios, and previous postpartum hemorrhage. These conditions may be associated with different baseline risks of postpartum hemorrhage, which should be considered when interpreting the pooled estimates. Therefore, the findings may not be directly generalizable to all high-risk cesarean populations, particularly to settings such as North America or Europe, where maternal risk profiles, obstetric practices, surgical protocols, and resource availability may differ.

Subgroup analyses were conducted to explore potential clinical and methodological sources of heterogeneity. These analyses did not identify statistically significant differences between subgroups. Although total blood loss was considered clinically comparable across trials, the very high heterogeneity indicates that the pooled estimate should be interpreted cautiously and should not be regarded as a definitive estimate of the magnitude of effect. Subgroup analyses according to placenta previa status were conducted when data were available; however, separate analyses for other risk categories were not feasible because these factors were inconsistently reported or overlapped within the same study populations. Meta-regression, funnel plot assessment, and Egger’s test were not performed because fewer than 10 studies were available per outcome, which precluded reliable interpretation of these analyses. Therefore, although publication bias and small-study effects could not be formally assessed, their possibility cannot be fully ruled out.

Despite these limitations, this study contributes to the available evidence by focusing specifically on high-risk women undergoing cesarean delivery, a population in whom clinical, pathophysiological, and obstetric risk factors for postpartum hemorrhage are particularly relevant. Further research on TXA in obstetrics is needed to optimize its efficacy and safety, determine the optimal dose, evaluate different regimens and routes of administration, and compare prophylactic and therapeutic approaches. Future multicenter trials should stratify participants by relevant risk factors and type of cesarean delivery to better assess treatment effects across clinically distinct subgroups. Additional studies may also explore long-term safety, effects on breastfeeding, and whether validated predictive tools or biomarkers could support more individualized prevention strategies for postpartum hemorrhage. These areas should be considered future research priorities rather than direct implications of the present findings.

## 5. Conclusions

Prophylactic TXA may reduce total blood loss in high-risk cesarean deliveries, although substantial heterogeneity was observed. A more homogeneous effect was observed in studies in which TXA was administered 15–20 min before skin incision.

TXA probably reduces intraoperative blood loss, the need for additional uterotonics, and blood transfusion. It may also reduce postpartum hemorrhage greater than 1000 mL and the need for complementary surgical interventions to control bleeding. Regarding hematological outcomes, TXA probably reduces the postoperative decrease in hemoglobin, whereas its effect on hematocrit remains uncertain.

Current evidence does not suggest an increased risk of adverse or serious adverse events, although the certainty of evidence for safety outcomes remains very low and the available studies remain underpowered to exclude rare thromboembolic complications.

## Figures and Tables

**Table 1:** Characteristics of included studies.

Author	Country	Study Design	Duration of Follow-Up	Number of Participants	Inclusion Criteria	Exclusion Criteria	Mean Age (SD)	Time of Application	Blood Loss Method	Diseases
Abdel-Fatah 2021 [16] and Abdel-Fatah 2022 [17]	Egypt	Randomized-controlled trial	Not reported	78	Pregnancy duration was between 35 and 42 weeks of gestation, elective cesarean delivery, women at risk of PPH, parity equal to or greater than 4, multiple pregnancies, uterine fibroids, previous postpartum hemorrhage, history of antepartum hemorrhage in the current pregnancy or previous pregnancies, anemia, fetal macrosomia, polyhydramnios	Severe medical and surgical complications involving the heart, liver, or kidney, brain disease, and blood disorders, bleeding tendency, Hypersensitivity to TXA, history of thromboembolic disorders	TXA group = 28.62 (6.0) Control group = 27.38 (7.1) *p*-value 0.411	10 min before skin incision	Not reported	Risk factors for PPH as multiple pregnancies, multiparous, previous PPH, history of antepartum hemorrhage in the current or previous pregnancy, anemia, fetal macrosomia, and polyhydramnios without a medical history of any chronic disease
Bhagat 2024 [18]	India	Double blind, xrandomized controlled trial	48 h	200	Gestation age between >x37 weeks and <42 weeks, age >18 years and <35 years, alive fetus, hemoglobin >x9 gm% in the recent third trimester, reports, subject xreceiving spinal anesthesia for C-section, women with high-risk pregnancies	Subjects with medical conditions such as kidney, heart, or liver disease that xcomplicate pregnancy, known cases of coagulation disorders, xintrauterine fetal death, undergoing anticoagulant treatment during the week before delivery, history of seizures, allergy to TXA or undergoing general anesthesia for a C-section	The majority of patients in both groups were aged 21–25 years, followed by those aged 26–30 years, with no xsignificant xdifference xbetween the groups xp-value 0.81	20 min xbefore skin incision	Gravimetric method	Pregnancy-induced hypertension (PIH), polyhydramnios, oligohydramnios, fetal distress, chorioamnionitis, history of C-section or PPH, twin pregnancy, prolonged and obstructed labor, prolonged induction, antepartum hemorrhage (APH)
Chaiyakarn 2023 [19]	Thailand	Double blind, randomized controlled trial	24 h	60	Women diagnosed with placenta previa by transabdominal or transvaginal ultrasound at 28 weeks or more of gestation, and cases undergoing C-section for placenta previa, either by elective C-section at 37 weeks or by emergency C-section due to placenta previa	Patients who show any signs of placental adhesion on the ultrasound scan prior to the procedure, patients with liver, heart or kidney disease, patients with TXA allergy and patients with risk factors for venous thromboembolism, such as thrombophilic disease, hematological cancer, immobilization or morbid obesity with a BMI greater than 40	TXA group = 34.7 (4.5) Control group = 32.7 (5.0) *p*-value 0.115	10 min before the skin incision	Gravimetric method	Placenta previa
Ifunanya 2019 [20]	Nigeria	Double blind, randomized controlled trial	6 weeks	168	Pregnant women who had at least one risk factor for PPH and who were to undergo elective or emergency cesarean delivery were eligible for inclusion in the study after obtaining informed consent	History of cardiac, renal, and liver diseases, bleeding disorders, history of any thrombogenic episodes, anticoagulant use and known allergy to TXA	TXA group = 28.2 (5.2) Control group = 28.6 (5.4) *p*-value 0.51	10 min before skin incision	Nadler formula	At least one of: hypertensive disorders in pregnancy, augmentation of labour, failed induction of labour, chorioamnionitis, placenta previa, placental abruption, polyhydramnios, multiparity, previous PPH, coexisting fibroids, fetal macrosomia, obstructed labour, multiple gestation and previous C-section
Jawad Iqbal 2022 [21]	Pakistan	Double blind, randomized placebo-controlled trial	6 weeks	60	Women with a scheduled elective C-section who were past 38 weeks of gestation and had at least one of the following risk factors: pregnancies >4, failed induction, obstructed or accelerated labor, placenta previa, chorioamnionitis, polyhydramnios, coexisting fibroids, fetal macrosomia, pregnancy-related hypertensive disorders and history of C-section or PPH	Women with other coexisting comorbidities such as cardiac, liver and renal disorders, bleeding disease with a history of anticoagulant use and allergic reaction to TXA	TXA group = 27.6 (4.3) Control group = 27.9 (4.7) *p*-value 0.61	Before skin incision	Nadler formula	Multiple gestations, failed induction, obstructed or augmentation of labor, placenta previa, chorioamnionitis, polyhydroamnious, coexisting fibroids, fetal macrosomia, pregnancy-related hypertensive disorders and a history of C-section or PPH
Mohamed 2024 [22]	Algeria	Double blind, randomized controlled trial	6 weeks	800	Aged between 18 and 50 years, with a live fetus and a gestational age greater than 35 weeks, presenting one or more risk factors for PPH, such as: age greater than 34 years, obesity, history of PPH or C-section, anemia, multiparity, uterine fibroid, preeclampsia, multifetal pregnancy, polyhydramnios, placenta previa, chorioamnionitis, macrosomia	Presence of renal failure, history of venous or arterial thrombosis, epilepsy, known allergy to TXA, gestational age less than 35 weeks, fetal death, placental insertion anomaly of the accreta type	TXA group = 34.2 (5.5) Control group = 33.6 (5.7) *p*-value 0.13	15 min before skin incision	Gravimetric method	Obesity, history of PPH, or C-section, anemia, multiparity, uterine fibroid, preeclampsia, multifetal pregnancy, polyhydramnios, placenta previa, chorioamniotitis, macrosomia
Nid Wongjariyakul 2025 [23]	Thailand	Double blind, randomized placebo-controlled trial	24 h	50	Pregnant women over the age of 18 at 34 weeks or more of gestation, with a scheduled C-section and undergoing spinal anesthesia, in addition, they had to have one or more risk factors for PPH	Women who had substantial medical conditions affecting the heart, liver, or kidneys, brain disorders, blood disorders, a known sensitivity to TXA, a history of or present venous or arterial thromboembolism, intrauterine fetal death or major fetal anomalies	Study group = 28.9 (5.0) Control group = 29.9 (5.2) *p*-value 0.479	10–15 min before skin incision	Gravimetric method	Previous cesarean delivery, fetal macrosomia, multiple gestation, low-lying placenta, polyhydramnios, placenta previa, prenatal anemia
Ortuanya 2024 [24]	Nigeria	Double blind, randomized controlled trial	48 h	200	High-risk pregnant women with a previous history of PPH, co-existing uterine fibroid, multiple pregnancy, previous C-section, fetal macrosomia, placenta previa, multiple gestation, severe preeclampsia, eclampsia and polyhydramnios	Participants who had uterine rupture, intrauterine fetal death, history of bleeding disorders, history of thromboembolism, significant antepartum hemorrhage, and known allergy to TXA, and unbooked patients with prolonged obstructed labor	Study group = 31.8 (4.0) Control group = 32.0 (4.7) *p*-value 0.09	10 min before surgery	EBL formula	Placenta previa, preeclampsia, eclampsia, fetal macrosomia, polyhydramnios, twin gestation with T1 breech, preterm premature rupture of membranes (PPROM), compound presentation, transverse, one or two previous C-sections
Shalaby 2022 [25]	Egypt	Double blind, randomized controlled trial	4 weeks	160	Women with elective C-section, aged between 20 and 40 years, and with a gestational age between 37 and 41 weeks, they had one or more risk factors for increased intraoperative blood loss, in addition, women with an overstretched uterus (e.g., multiple gestation, macrosomic fetus, or polyhydramnios), placenta previa, anemia, and those who had received an intraoperative blood transfusion during a previous C-section	Women with a history of thromboembolic events, allergy to TXA and those with a morbidly adherent placenta, women who were expected to experience intraoperative complications, such as visceral injuries	TXA group = 28.9 (4.46) Control group = 28.5 (4.45) *p*-value 0.758	15 min before surgery	EBL formula and gravimetric method	Anemia, polyhydramnios, fetal macrosomia, twin pregnancy, placenta previa and received blood transfusion during previous C-section

Abbreviations: PPH, postpartum hemorrhage; TXA, tranexamic acid; SD, standard deviation; NR, not reported; RCT, randomized controlled trial. Notes: Mean age is presented as mean (SD). Timing of the intervention refers to TXA administration relative to skin incision. Blood loss estimation methods were reported as described in the original studies.

## Data Availability

The data used in this systematic review and meta-analysis were derived from published studies. All relevant data are included in the article and its Supplementary Materials (search strategy, GRADE assessment, and supplementary meta-analyses).

## Supplementary Materials

The following supporting information can be downloaded at https://www.mdpi.com/article/10.3390/jcm15124630/s1, Table S1. Search strategy; Table S2. GRADE assessment for primary outcomes; Table S3. GRADE assessment for secondary outcomes; Table S4. PRISMA 2020 Checklist; Table S5. Handling of overlapping publications from the Abdel-Fatah trial; Figure S1. Sensitivity analysis of total blood loss excluding Bhagat 2024; Figure S2; Subgroup analysis of total blood loss by blood loss quantification method; Figure S3; Subgroup analysis of total blood loss by placenta previa status; Figure S4. Subgroup analysis of total blood loss by geographic region; Figure S5. Subgroup analysis of total blood loss by sample size; Figure S6. Subgroup analysis of total blood loss by type of cesarean delivery; Figure S7. Subgroup analysis of total blood loss by mean maternal age; Figure S8. Subgroup analysis of total blood loss by timing of tranexamic acid administration; Figure S9. Meta-analysis of total blood loss; Figure S10. Subgroup analysis of total blood loss by timing of tranexamic acid administration; Figure S11. Subgroup analysis of total blood loss by blood loss quantification method; Figure S12. Subgroup analysis of total blood loss by placenta previa status; Figure S13. Subgroup analysis of total blood loss by geographic region; Figure S14. Subgroup analysis of total blood loss by sample size; Figure S15. Subgroup analysis of total blood loss by type of cesarean delivery; Figure S16. Subgroup analysis of total blood loss by mean maternal age; Figure S17. Sensitivity analysis for intraoperative blood loss; Figure S18. Meta-analysis of blood loss 2 h postpartum; Figure S19. Baujat plot for postoperative hemoglobin using mean difference; Figure S20. Leave-one-out heterogeneity analysis for postoperative hemoglobin using mean difference; Figure S21. Meta-analysis of hospital length of stay using the mean difference; Figure S22. Meta-analysis of additional surgical intervention; Figure S23. Meta-analysis of side effects; Figure S24. Meta-analysis of serious adverse events.
