# Supplementary material for: Prophylactic Use of Tranexamic Acid to Prevent Postpartum Hemorrhage in High-Risk Cesarean Deliveries: A Systematic Review and Meta-Analysis
Source: J Clin Med. 2026 Jun 15;15(12):4630. doi: 10.3390/jcm15124630 (PMC13301122; doi:10.3390/jcm15124630)

Title: Prophylactic use of tranexamic acid to prevent postpartum hemorrhage in high-risk cesarean deliveries: a systematic review and meta-analysis

Supplementary material—Content

Table S1. Search strategy.
Table S2. GRADE assessment for primary outcomes.
Table S3. GRADE assessment for secondary outcomes.
Table S4. PRISMA 2020 Checklist.
Table S5. Handling of overlapping publications from the Abdel-Fatah trial.
Figure S1. Sensitivity analysis of total blood loss excluding Bhagat 2024.
Figure S2. Subgroup analysis of total blood loss by blood loss quantification method.
Figure S3. Subgroup analysis of total blood loss by placenta previa status.
Figure S4. Subgroup analysis of total blood loss by geographic region.
Figure S5. Subgroup analysis of total blood loss by sample size.
Figure S6. Subgroup analysis of total blood loss by type of cesarean delivery.
Figure S7. Subgroup analysis of total blood loss by mean maternal age.
Figure S8. Subgroup analysis of total blood loss by timing of tranexamic acid administration.
Figure S9. Meta-analysis of total blood loss.
Figure S10. Subgroup analysis of total blood loss by timing of tranexamic acid administration.
Figure S11. Subgroup analysis of total blood loss by blood loss quantification method.
Figure S12. Subgroup analysis of total blood loss by placenta previa status.
Figure S13. Subgroup analysis of total blood loss by geographic region.
Figure S14. Subgroup analysis of total blood loss by sample size.
Figure S15. Subgroup analysis of total blood loss by type of cesarean delivery.
Figure S16. Subgroup analysis of total blood loss by mean maternal age.
Figure S17. Sensitivity analysis for intraoperative blood loss.
Figure S18. Meta-analysis of blood loss 2 h postpartum.
Figure S19. Baujat plot for postoperative hemoglobin using mean difference.
Figure S20. Leave-one-out heterogeneity analysis for postoperative hemoglobin using mean difference.
Figure S21. Meta-analysis of hospital length of stay using the mean difference.
Figure S22. Meta-analysis of additional surgical intervention.
Figure S23. Meta-analysis of side effects.
Figure S24. Meta-analysis of serious adverse events.

Table S1. Search strategy.

SEARCH ENGINE	STRATEGY	RESULTS
PUBMED	("Cesarean Section" OR "Cesarean Sections" OR "Abdominal Delivery" OR "C-Section (OB)" OR "C Section (OB)" OR "C-Sections (OB)" OR "Caesarean Section" OR "Caesarean Sections" OR "Delivery, Abdominal" OR "Abdominal Deliveries" OR "Deliveries, Abdominal" OR "Postcesarean Section") AND ("Tranexamic Acid" OR "AMCA" OR "AMCHA" OR "t-AMCHA" OR "trans-4-(Aminomethyl)cyclohexanecarboxylic Acid" OR "Cyklokapron" OR "Ugurol" OR "Transamin" OR "KABI 2161" OR "Amchafibrin" OR "Anvitoff" OR "Spotof" OR "Exacyl") AND ("Postpartum Hemorrhage" OR "Hemorrhage, Postpartum" OR "Delayed Postpartum Hemorrhage" OR "Hemorrhage, Delayed Postpartum" OR "Postpartum Hemorrhage, Delayed" OR "Immediate Postpartum Hemorrhage" OR "Hemorrhage, Immediate Postpartum" OR "Postpartum Hemorrhage, Immediate")	128
SCOPUS	(TITLE-ABS-KEY ("Cesarean Section" OR "Cesarean Sections" OR "Abdominal Delivery" OR "C-Section (OB)" OR "C Section (OB)" OR "C-Sections (OB)" OR "Caesarean Section" OR "Caesarean Sections" OR "Delivery, Abdominal" OR "Abdominal Deliveries" OR "Deliveries, Abdominal" OR "Postcesarean Section")) AND (TITLE-ABS-KEY ("Tranexamic Acid" OR "AMCA" OR "AMCHA" OR "t-AMCHA" OR "trans-4-(Aminomethyl)cyclohexanecarboxylic Acid" OR "Cyklokapron" OR "Ugurol" OR "Transamin" OR "KABI 2161" OR "Amchafibrin" OR "Anvitoff" OR "Spotof" OR "Exacyl")) AND (TITLE-ABS-KEY ("Postpartum Hemorrhage" OR "Hemorrhage, Postpartum" OR "Delayed Postpartum Hemorrhage" OR "Hemorrhage, Delayed Postpartum" OR "Postpartum Hemorrhage, Delayed" OR "Immediate Postpartum Hemorrhage" OR "Hemorrhage, Immediate Postpartum" OR "Postpartum Hemorrhage, Immediate"))	538
WEB OF SCIENCE	TS=("cesarean section*" OR "caesarean section*" OR "c-section" OR "abdominal delivery*")<br>AND<br>TS=("tranexamic acid" OR "cyklokapron" OR "transamin" OR "exacyl")<br>AND<br>TS=("postpartum hemorrhag*" OR "postpartum bleeding" OR "postpartum blood loss" OR "postpartum haemorrhag*")	282

Embase	("Cesarean Section" OR "Cesarean Sections" OR "Abdominal Delivery" OR "C-Section (OB)" OR "C Section (OB)" OR "C-Sections (OB)" OR "Caesarean Section" OR "Caesarean Sections" OR "Delivery, Abdominal" OR "Abdominal Deliveries" OR "Deliveries, Abdominal" OR "Postcesarean Section"):ti,ab,kw,de<br>AND<br>("Tranexamic Acid" OR "AMCA" OR "AMCHA" OR "t-AMCHA" OR "trans-4-(Aminomethyl)cyclohexanecarboxylic Acid" OR "Cyklokapron" OR "Ugurol" OR "Transamin" OR "KABI 2161" OR "Amchafibrin" OR "Anvitoff" OR "Spotof" OR "Exacyl"):ti,ab,kw,de<br>AND<br>("Postpartum Hemorrhage" OR "Hemorrhage, Postpartum" OR "Delayed Postpartum Hemorrhage" OR "Hemorrhage, Delayed Postpartum" OR "Postpartum Hemorrhage, Delayed" OR "Immediate Postpartum Hemorrhage" OR "Hemorrhage, Immediate Postpartum" OR "Postpartum Hemorrhage, Immediate"):ti,ab,kw,de	701
Cochrane Library<br><br>Embase: 85<br>Pubmed: 49<br>ClinicalTrials.gov: 28<br>ICTRP (International Clinical Trials Registry Platform): 26<br>CINAHL: 2	("cesarean section" OR "cesarean sections" OR "abdominal delivery" OR "c-section" OR "caesarean section" OR "caesarean sections" OR "delivery, abdominal" OR "postcesarean section")<br>AND<br>("tranexamic acid" OR "amca" OR "amcha" OR "t-amcha" OR "trans-4-(aminomethyl)cyclohexanecarboxylic acid" OR "cyklokapron" OR "ugurol" OR "transamin" OR "kabi 2161" OR "amchafibrin" OR "anvitoff" OR "spotof" OR "exacyl")<br>AND<br>("postpartum hemorrhage" OR "hemorrhage, postpartum" OR "delayed postpartum hemorrhage" OR "hemorrhage, delayed postpartum" OR "immediate postpartum hemorrhage" OR "hemorrhage, immediate postpartum")	162

Table S2. GRADE assessment for primary outcomes.

Question: Tranexamic acid compared to placebo for to prevent postpartum hemorrhage in high-risk cesarean deliveries

Certainty assessment							№ of patients		Effect		Certainty	Importance
№ of studies	Study design	Risk of bias	Inconsistency	Indirectness	Imprecision	Other considerations	Tranexamic acid	Placebo	Relative (95% CI)	Absolute (95% CI)		
**Total blood loss (ml) (assessed with: ml)**												
6	randomised trials	serious[a]	serious[b]	not serious	not serious	strong association	433	433	-	MD **308.78 ml lower** (459.78 lower to 157.77 lower)	⊕⊕⊕◯ Moderate[a,b]	
**Total blood loss - Time grouped 10 min (assessed with: ml)**												
4	randomised trials	serious[a]	very serious[b]	not serious	not serious	strong association	253	253	-	SMD **2.31 SD lower** (3.5 lower to 1.13 lower)	⊕⊕◯◯ Low[a,b]	
**Total blood loss - Time grouped 15-20 min (assessed with: ml)**												
2	randomised trials	serious[c]	not serious	not serious	not serious	none	180	180	-	SMD **0.61 SD lower** (0.82 lower to 0.39 lower)	⊕⊕⊕◯ Moderate[c]	
**Intraoperative blood loss (assessed with: ml)**												
4	randomised trials	serious[a]	serious[b]	not serious	not serious	strong association	494	494	-	MD **256.71 ml lower** (375.04 lower to 138.39 lower)	⊕⊕⊕◯ Moderate[a,b]	
**Blood loss 2 hours (assessed with: ml)**												
2	randomised trials	serious[c]	very serious[b]	not serious	not serious	none	500	500	-	MD **21.21 ml lower** (53.15 lower to 10.74 higher)	⊕◯◯◯ Very low[b,c]	
**Blood loss > 1000 ml (assessed with: ml)**												

Certainty assessment							№ of patients		Effect		Certainty	Importance
№ of studies	Study design	Risk of bias	Inconsistency	Indirectness	Imprecision	Other considerations	Tranexamic acid	Placebo	Relative (95% CI)	Absolute (95% CI)		
6	randomised trials	serious[d]	not serious	not serious	serious[e]	strong association	22/669 (3.3%)	108/669 (16.1%)	**RR 0.24** (0.14 to 0.41)	**123 fewer per 1,000** (from 139 fewer to 95 fewer)	⊕⊕⊕◯ Moderate[d,e]	

CI: confidence interval; **MD:** mean difference; **RR:** risk ratio; **SMD:** standardised mean difference

Explanations

a. Downgraded one level due to some concerns in the deviations from the intended interventions, measurement of the outcome and selection of the reported results.
b. Downgraded one level because one of the study no overlap
c. Downgraded one level due to some concerns in the selection of the reported results.
d. Downgraded one lievel due to some concerns in the randomisation process, deviations from the intended interventions and selection of the reported results
e. Downgraded one level due to crosses the clinical decision threshold

Table S3. GRADE assessment for secondary outcomes.

Question: Tranexamic acid compared to placebo for to prevent postpartum hemorrhage in high-risk cesarean deliveries

Certainty assessment							№ of patients		Effect		Certainty	Importance
№ of studies	Study design	Risk of bias	Inconsistency	Indirectness	Imprecision	Other considerations	Tranexamic acid	Placebo	Relative (95% CI)	Absolute (95% CI)		
**Hemoglobin post (assessed with: g/dL)**												
7	randomised trials	serious[a]	serious[b]	not serious	not serious	strong association	834	834	-	MD **1.63 g/dL higher** (0.5 higher to 2.75 higher)	⊕⊕⊕◯ Moderate[a,b]	
**Hematocrit post (assessed with: %)**												
4	randomised trials	serious[a]	serious[c]	not serious	serious[d]	strong association	249	249	-	MD **1.83 % higher** (0.6 higher to 3.06 higher)	⊕⊕◯◯ Low[a,c,d]	
**Hospital stay (assessed with: days)**												

Certainty assessment							№ of patients		Effect		Certainty	Importance
№ of studies	Study design	Risk of bias	Inconsistency	Indirectness	Imprecision	Other considerations	Tranexamic acid	Placebo	Relative (95% CI)	Absolute (95% CI)		
3	randomised trials	serious[e]	not serious	not serious	extremely serious[d]	none	455	455	-	MD **0.02 days higher** (0.1 lower to 0.14 higher)	⨁◯◯◯ Very low[d,e]	
**Additional uterotonics**												
7	randomised trials	serious[f]	serious[g]	not serious	serious[d]	strong association	90/749 (12.0%)	222/749 (29.6%)	**RR 0.37** (0.24 to 0.58)	**187 fewer per 1,000** (from 225 fewer to 124 fewer)	⨁⨁◯◯ Low[d,f,g]	
**Surgical intervention**												
2	randomised trials	serious[f]	not serious	not serious	serious[d]	none	7/130 (5.4%)	20/130 (15.4%)	**RR 0.35** (0.16 to 0.78)	**100 fewer per 1,000** (from 129 fewer to 34 fewer)	⨁⨁◯◯ Low[d,f]	
**Blood transfusion need**												
8	randomised trials	serious[e]	not serious	not serious	very serious[d]	strong association	25/849 (2.9%)	90/849 (10.6%)	**RR 0.30** (0.22 to 0.40)	**74 fewer per 1,000** (from 83 fewer to 64 fewer)	⨁⨁◯◯ Low[d,e]	
**Side effects**												
3	randomised trials	serious[f]	very serious[b]	not serious	extremely serious[d]	none	86/525 (16.4%)	20/525 (3.8%)	**RR 3.47** (0.65 to 18.41)	**94 more per 1,000** (from 13 fewer to 663 more)	⨁◯◯◯ Very low[b,d,f]	
**Serious events**												
3	randomised trials	serious[f]	not serious	not serious	extremely serious[d]	none	4/530 (0.8%)	4/530 (0.8%)	**RR 1.00** (0.29 to 3.43)	**0 fewer per 1,000** (from 5 fewer to 18 more)	⨁◯◯◯ Very low[d,f]	

CI: confidence interval; **MD:** mean difference; **RR:** risk ratio

Explanations

a. Downgraded one level due to some concerns in the deviations from the intended interventions, measurement of the outcome and selection of the reported results.
b. Downgraded one level because the studies not overlap and effect size is different
c. Downgraded one level because the studies not overlap
d. Downgraded one level due to crosses the clinical decision threshold
e. Downgraded one level due to some concerns in the selection of the reported results.
f. Downgraded one level due to some concerns in the randomisation process, deviations from the intended interventions and selection of the reported results.
g. Downgraded one level due to moderate heterogeneity

Table S4. PRISMA 2020 Checklist.

Section and Topic	Item #	Checklist item	Location where item is reported
**TITLE**			
Title	1	Identify the report as a systematic review.	Page 1
**ABSTRACT**			
Abstract	2	See the PRISMA 2020 for Abstracts checklist.	Page 1
**INTRODUCTION**			
Rationale	3	Describe the rationale for the review in the context of existing knowledge.	Pages 1–2
Objectives	4	Provide an explicit statement of the objective(s) or question(s) the review addresses.	Page 2
**METHODS**			
Eligibility criteria	5	Specify the inclusion and exclusion criteria for the review and how studies were grouped for the syntheses.	Pages 2–3
Information sources	6	Specify all databases, registers, websites, organisations, reference lists and other sources searched or consulted to identify studies. Specify the date when each source was last searched or consulted.	Page 2
Search strategy	7	Present the full search strategies for all databases, registers and websites, including any filters and limits used.	Supplementary material, Table S1
Selection process	8	Specify the methods used to decide whether a study met the inclusion criteria of the review, including how many reviewers screened each record and each report retrieved, whether they worked independently, and if applicable, details of automation tools used in the process.	Page 3
Data collection process	9	Specify the methods used to collect data from reports, including how many reviewers collected data from each report, whether they worked independently, any processes for obtaining or confirming data from study investigators, and if applicable, details of automation tools used in the process.	Page 3
Data items	10a	List and define all outcomes for which data were sought. Specify whether all results that were compatible with each outcome domain in each study were sought (e.g. for all measures, time points, analyses), and if not, the methods used to decide which results to collect.	Page 3
	10b	List and define all other variables for which data were sought (e.g. participant and intervention	Page 3

		characteristics, funding sources). Describe any assumptions made about any missing or unclear information.	
Study risk of bias assessment	11	Specify the methods used to assess risk of bias in the included studies, including details of the tool(s) used, how many reviewers assessed each study and whether they worked independently, and if applicable, details of automation tools used in the process.	Page 3
Effect measures	12	Specify for each outcome the effect measure(s) (e.g. risk ratio, mean difference) used in the synthesis or presentation of results.	Pages 3–4
Synthesis methods	13a	Describe the processes used to decide which studies were eligible for each synthesis (e.g. tabulating the study intervention characteristics and comparing against the planned groups for each synthesis (item #5)).	Pages 2–4
	13b	Describe any methods required to prepare the data for presentation or synthesis, such as handling of missing summary statistics, or data conversions.	Pages 3–4
	13c	Describe any methods used to tabulate or visually display results of individual studies and syntheses.	Pages 6 and 11–15; Figures 1–7; Supplementary Figures S1–S24
	13d	Describe any methods used to synthesize results and provide a rationale for the choice(s). If meta-analysis was performed, describe the model(s), method(s) to identify the presence and extent of statistical heterogeneity, and software package(s) used.	Pages 3–4
	13e	Describe any methods used to explore possible causes of heterogeneity among study results (e.g. subgroup analysis, meta-regression).	Pages 3–4 and 11–12
	13f	Describe any sensitivity analyses conducted to assess robustness of the synthesized results.	Pages 3–4 and 11–13
Reporting bias assessment	14	Describe any methods used to assess risk of bias due to missing results in a synthesis (arising from reporting biases).	Page 4
Certainty assessment	15	Describe any methods used to assess certainty (or confidence) in the body of evidence for an outcome.	Page 4
**RESULTS**			
Study selection	16a	Describe the results of the search and selection process, from the number of records identified in the search to the number of studies included in the review, ideally using a flow diagram.	Pages 4–6 and Figure 1
	16b	Cite studies that might appear to meet the inclusion	Page 4

		criteria, but which were excluded, and explain why they were excluded.	
Study characteristics	17	Cite each included study and present its characteristics.	Pages 5–10 and Table 1
Risk of bias in studies	18	Present assessments of risk of bias for each included study.	Page 11 and Figure 2
Results of individual studies	19	For all outcomes, present, for each study: (a) summary statistics for each group (where appropriate) and (b) an effect estimate and its precision (e.g. confidence/credible interval), ideally using structured tables or plots.	Pages 11–15 and Figures 3–7; Supplementary Figures S1–S24
Results of syntheses	20a	For each synthesis, briefly summarise the characteristics and risk of bias among contributing studies.	Pages 11–15
	20b	Present results of all statistical syntheses conducted. If meta-analysis was done, present for each the summary estimate and its precision (e.g. confidence/credible interval) and measures of statistical heterogeneity. If comparing groups, describe the direction of the effect.	Pages 11–15 and Figures 3–7; Supplementary Figures S1–S24
	20c	Present results of all investigations of possible causes of heterogeneity among study results.	Pages 11–12; Supplementary Figures S1–S16
	20d	Present results of all sensitivity analyses conducted to assess the robustness of the synthesized results.	Pages 11–13; Supplementary Figures S1 and S17–S20
Reporting biases	21	Present assessments of risk of bias due to missing results (arising from reporting biases) for each synthesis assessed.	Pages 4 and 17. Not formally assessed because each synthesis included fewer than 10 studies, limiting the reliability of funnel plot asymmetry tests and Egger's test.
Certainty of evidence	22	Present assessments of certainty (or confidence) in the body of evidence for each outcome assessed.	Pages 11–18; Supplementary Tables S2–S3 (GRADE)
**DISCUSSION**			
Discussion	23a	Provide a general interpretation of the results in the context of other evidence.	Pages 15–17
	23b	Discuss any limitations of the evidence included in the review.	Page 17

	23c	Discuss any limitations of the review processes used.	Page 17
	23d	Discuss implications of the results for practice, policy, and future research.	Pages 17–18
**OTHER INFORMATION**			
Registration and protocol	24a	Provide registration information for the review, including register name and registration number, or state that the review was not registered.	Page 2
	24b	Indicate where the review protocol can be accessed, or state that a protocol was not prepared.	Page 2
	24c	Describe and explain any amendments to information provided at registration or in the protocol.	No amendments to the registered protocol were made.
Support	25	Describe sources of financial or non-financial support for the review, and the role of the funders or sponsors in the review.	Page 18
Competing interests	26	Declare any competing interests of review authors.	Page 18
Availability of data, code and other materials	27	Report which of the following are publicly available and where they can be found: template data collection forms; data extracted from included studies; data used for all analyses; analytic code; any other materials used in the review.	Page 18 and Supplementary material

From: Page MJ, McKenzie JE, Bossuyt PM, Boutron I, Hoffmann TC, Mulrow CD, et al. The PRISMA 2020 statement: an updated guideline for reporting systematic reviews. BMJ 2021;372:n71. doi: 10.1136/bmj.n71. This work is licensed under CC BY 4.0. To view a copy of this license, visit https://creativecommons.org/licenses/by/4.0

Table S5. Handling of overlapping publications from the Abdel-Fatah trial.

Study group	Publication	Authors	Evidence of overlap	Specific outcomes used in the meta-analysis	Handling decision
Abdel-Fatah *et al.*	Abdel-Fatah et al., 2021. *Evaluation of the efficacy of tranexamic acid in preventing postpartum haemorrhage in high risk patients delivered by CS.* Journal of Cardiovascular Disease Research.	Ashraf Talat Abdel-Fatah, Basem Mohamed Hamed, Sadina Ahmad Ali Mohammed, Safaa Abdel-Salam Ibrahim	Same author group, same institution, same study period, same sample size, same treatment allocation, same TXA regimen, same comparator, and same main blood loss estimates as the 2022 publication. The study included 78 women, with 39 in the TXA group and 39 in the control group. TXA was administered as 1 g intravenously 10 minutes before skin incision, and the comparator was 10 mL normal saline.	Used as the primary source for the following outcomes when data were available: total/intraoperative blood loss during cesarean section, postoperative hemoglobin, postoperative hematocrit, need for blood transfusion, need for additional uterotonics or medical interventions, need for surgical interventions to control bleeding, and maternal/neonatal adverse events.	Treated as the more complete report of the randomized trial. Outcome data were extracted only once for each outcome.
Abdel-Fatah *et al.*	Abdel-Fatah et al., 2022. *Effectiveness of Tranexamic Acid in Preventing Postpartum Hemorrhage in Cesarean Delivery of High-Risk Pregnancy.* The Egyptian Journal of Hospital Medicine.	Ashraf Talat Abdel-Fatah, Safaa Abdel-Salam Ibrahim, Sadina Ahmad Ali Mohammed, Basem Mohamed Hamed	Same author group, same institution, same study period, same sample size, same treatment allocation, same TXA regimen, same comparator, and same main blood loss estimates. The study included 78 women, with 39 in the TXA group and 39 in the control group. TXA was administered as 1 g intravenously 10 minutes before skin incision, and the comparator was 10 mL normal saline.	Used only to verify duplicated information, including sample size, baseline characteristics, risk factors for postpartum hemorrhage, TXA timing and dose, comparator, and blood loss during cesarean section.	Treated as a companion report of the same randomized trial. It was not counted as an independent study in any meta-analysis.
Abdel-Fatah *et al.* 2021/2022	Multiple reports of the same randomized trial	Same author group across both reports	Both publications reported the same trial population and overlapping outcome data.	The following duplicated or potentially overlapping outcomes were extracted once only: blood loss during cesarean section, hemoglobin, hematocrit, blood transfusion, additional uterotonics/medical interventions, surgical interventions, and adverse events, when reported.	No double counting was performed. When the same outcome was reported in both publications, the more complete or more detailed report was prioritized.

Note: TXA, tranexamic acid. Abdel-Fatah et al. 2021 and Abdel-Fatah et al. 2022 were treated as companion reports of the same randomized trial, and participant data were included only once per outcome to avoid double counting.

Figure S1. Sensitivity analysis of total blood loss excluding Bhagat 2024.

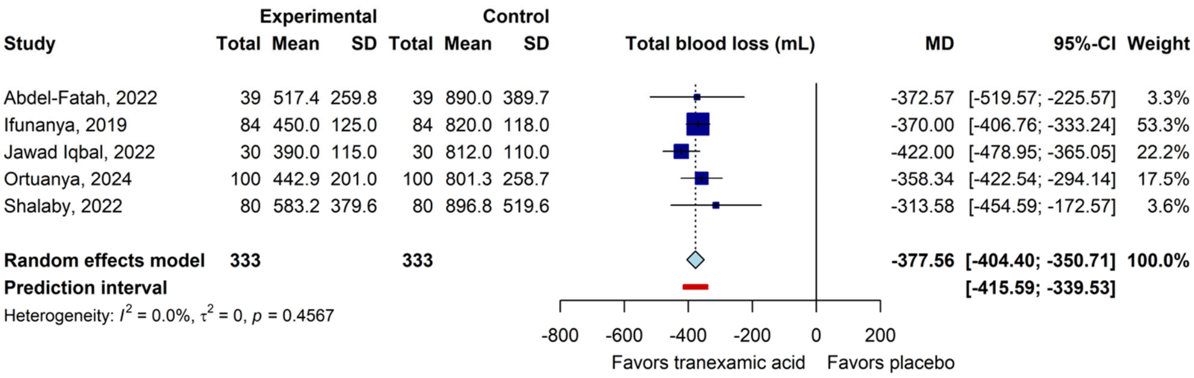

Figure S2. Subgroup analysis of total blood loss by blood loss quantification method.

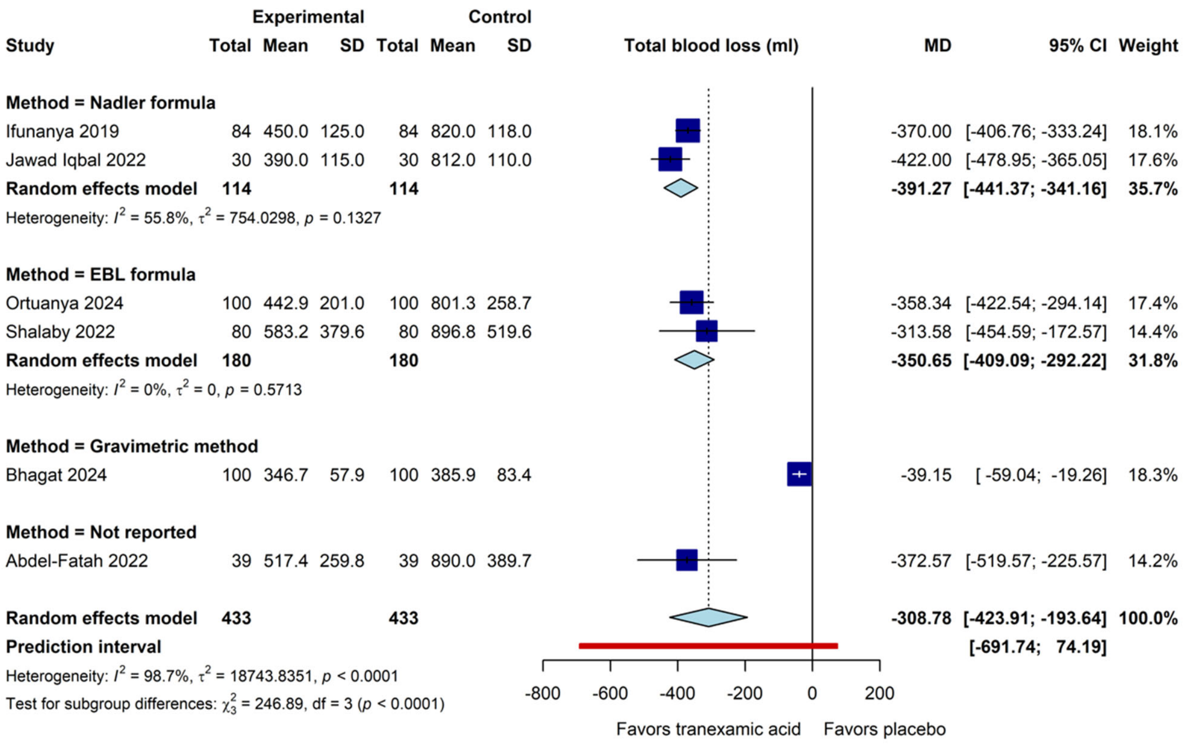

Figure S3. Subgroup analysis of total blood loss by placenta previa status.

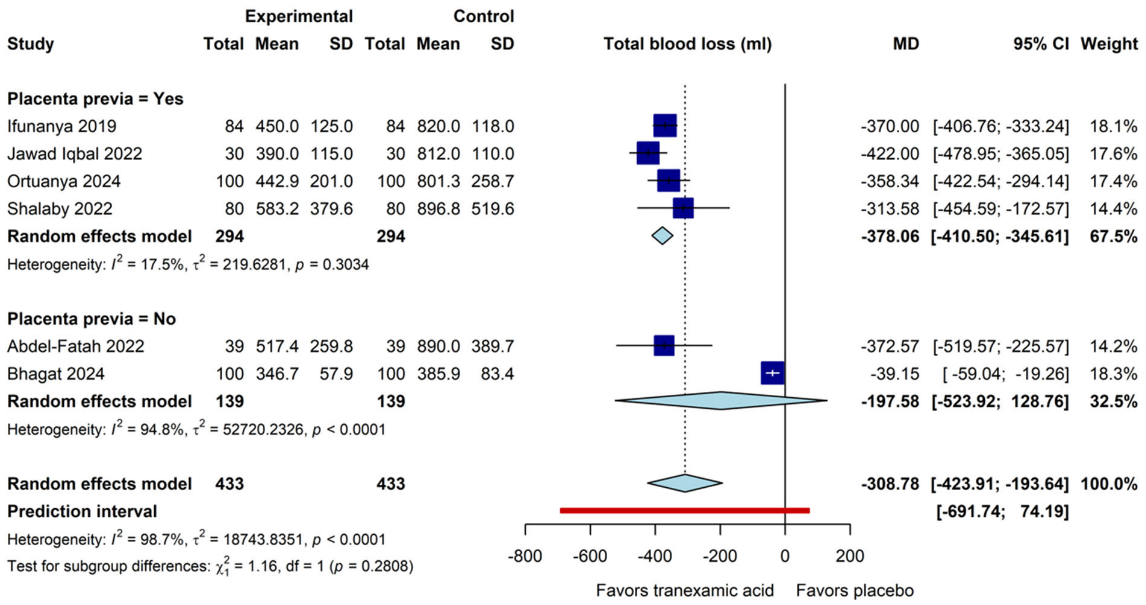

Figure S4. Subgroup analysis of total blood loss by geographic region.

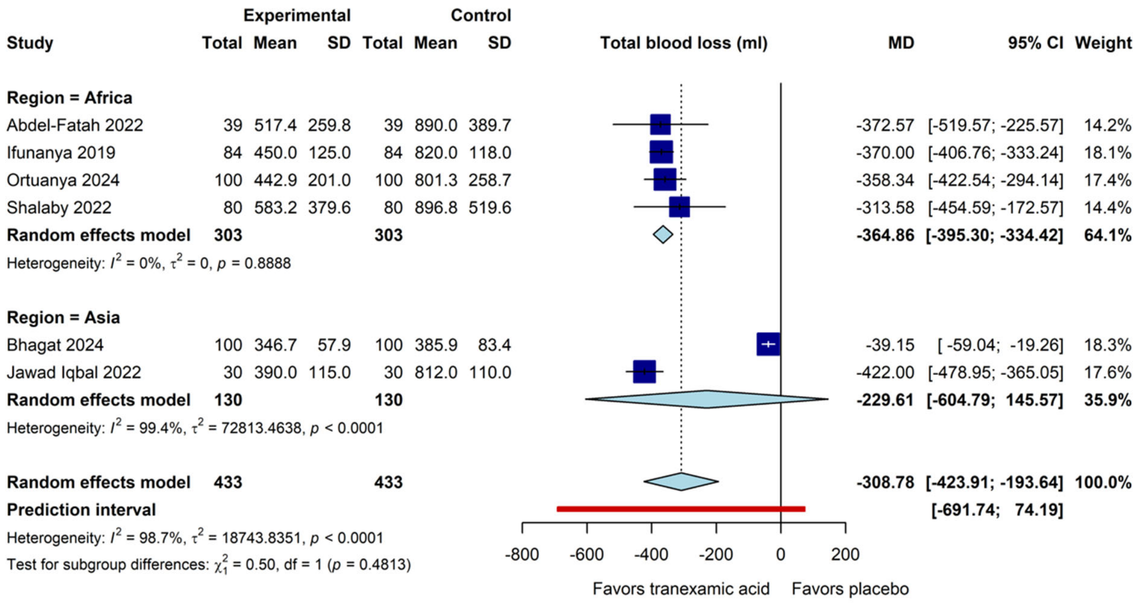

Figure S5. Subgroup analysis of total blood loss by sample size.

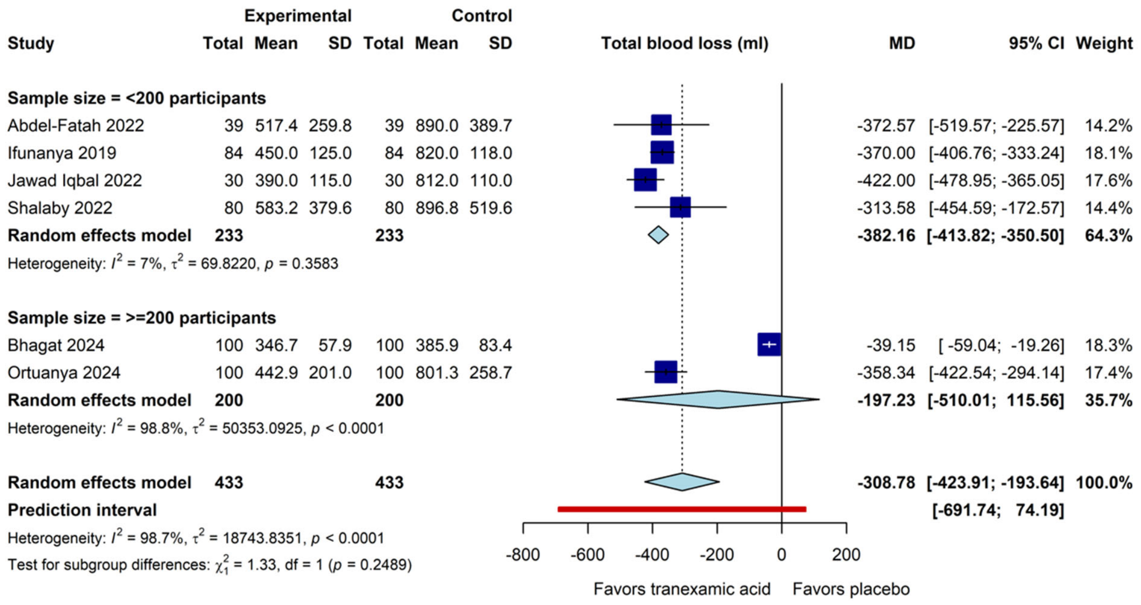

Figure S6. Subgroup analysis of total blood loss by type of cesarean delivery.

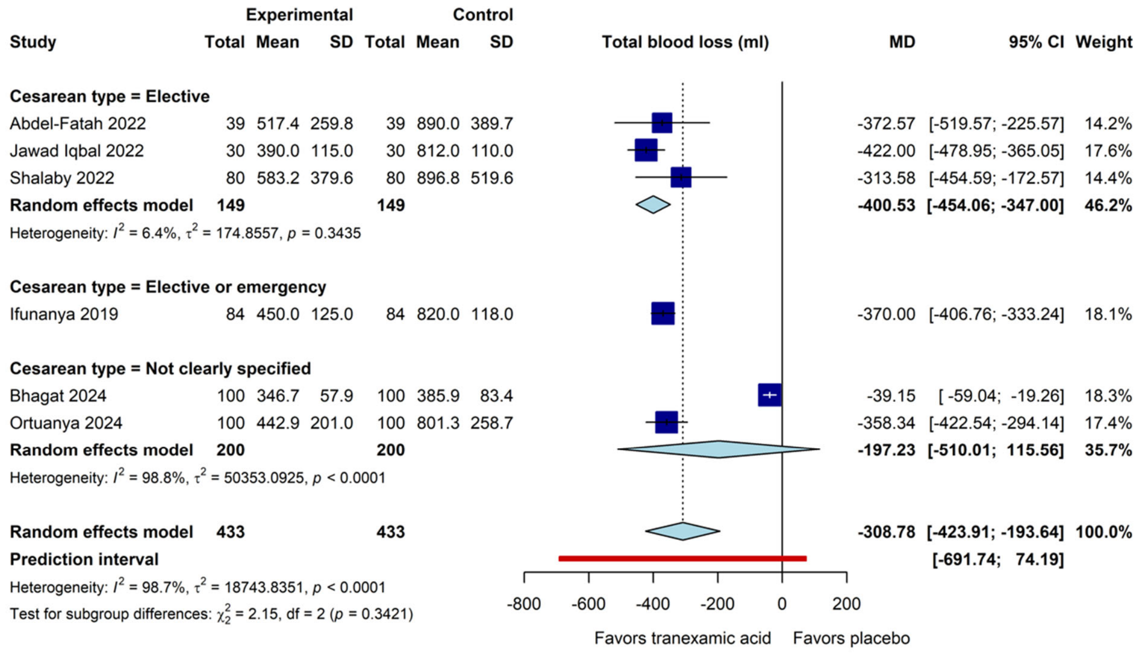

Figure S7. Subgroup analysis of total blood loss by mean maternal age.

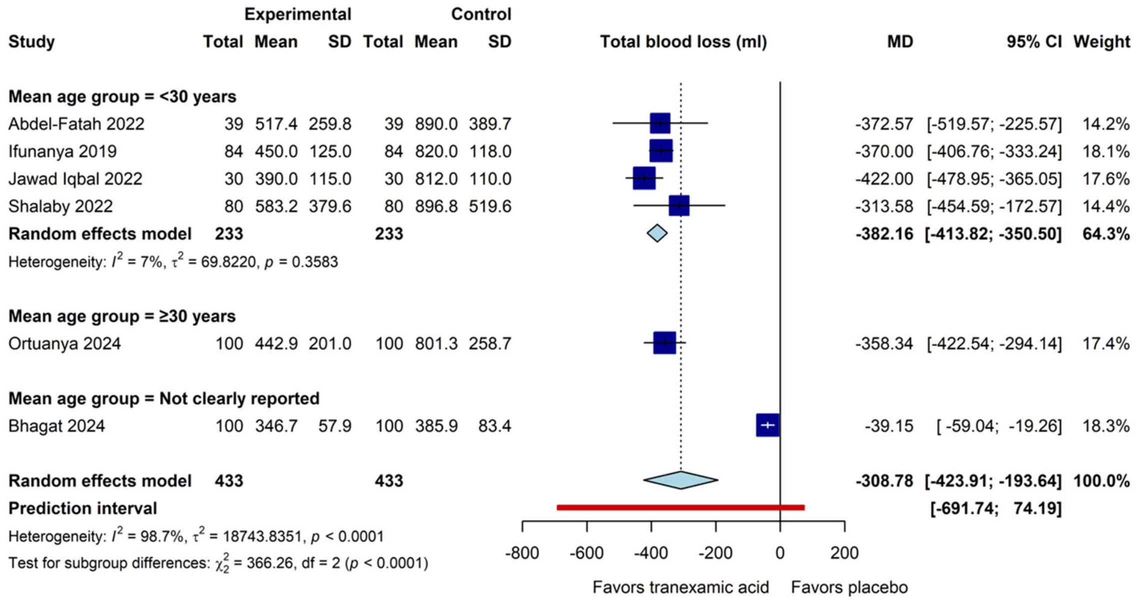

Figure S8. Subgroup analysis of total blood loss by timing of tranexamic acid administration.

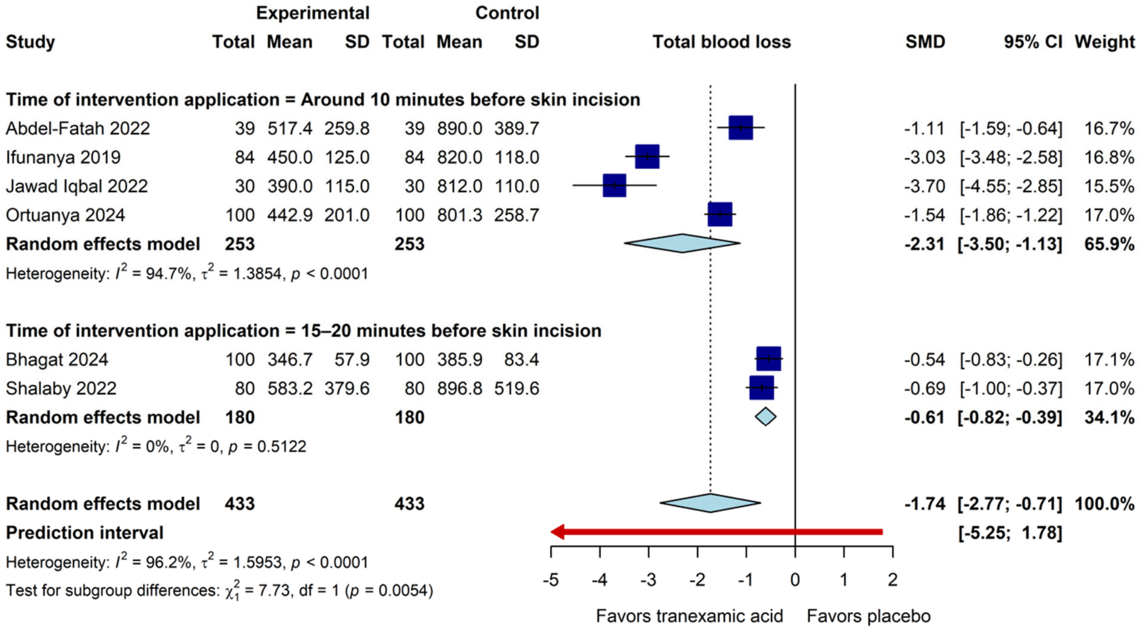

Figure S9. Meta-analysis of total blood loss.

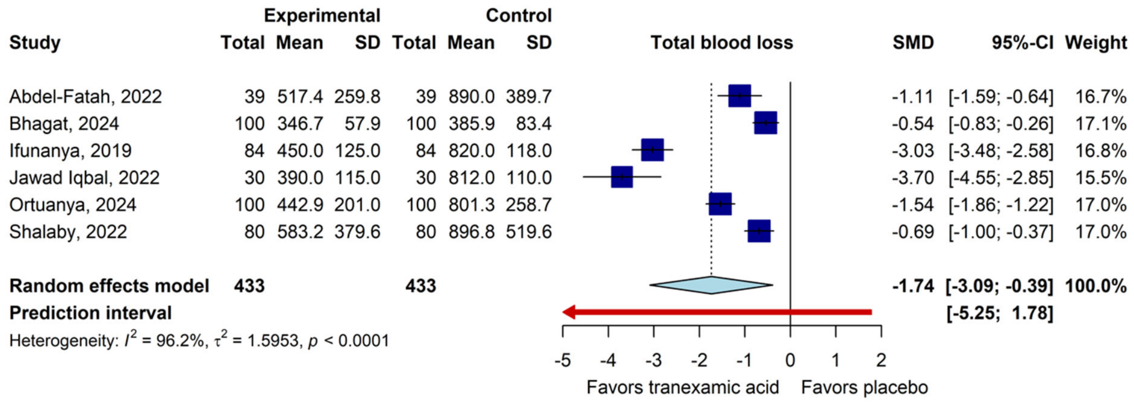

Figure S10. Subgroup analysis of total blood loss by timing of tranexamic acid administration.

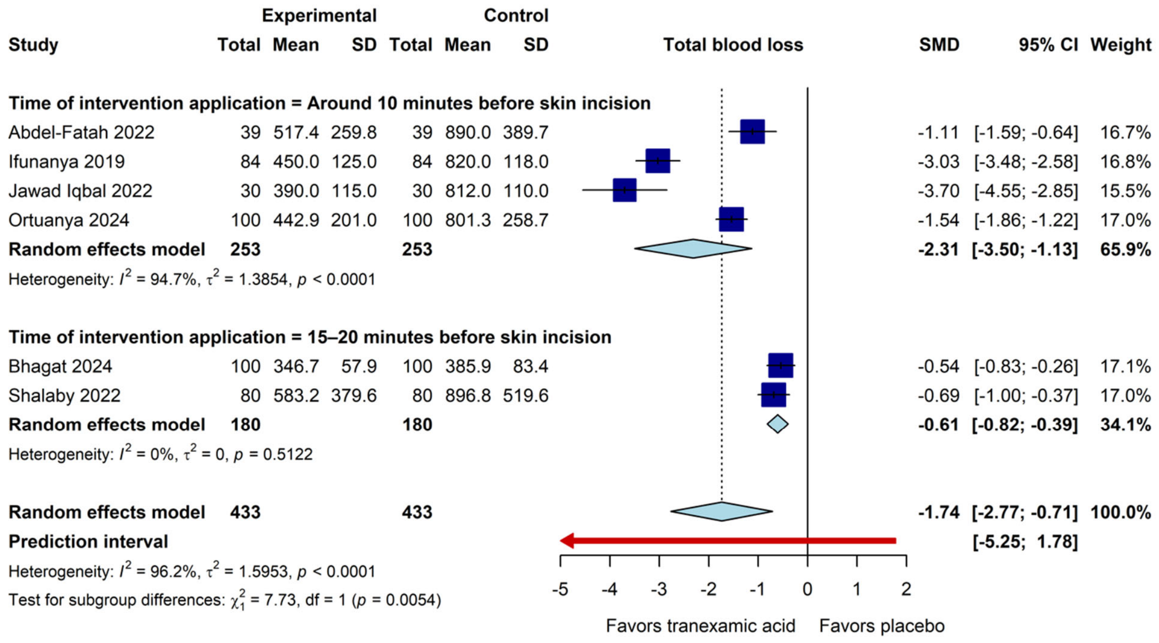

Figure S11. Subgroup analysis of total blood loss by blood loss quantification method.

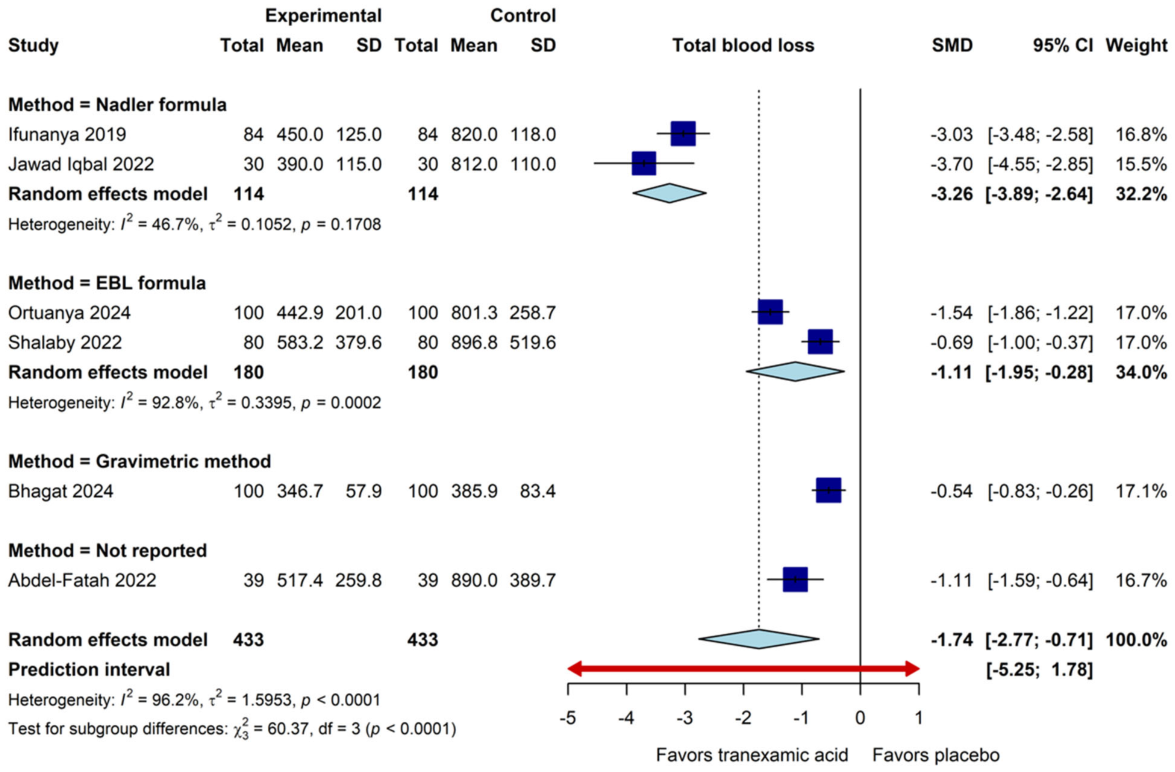

Figure S12. Subgroup analysis of total blood loss by placenta previa status.

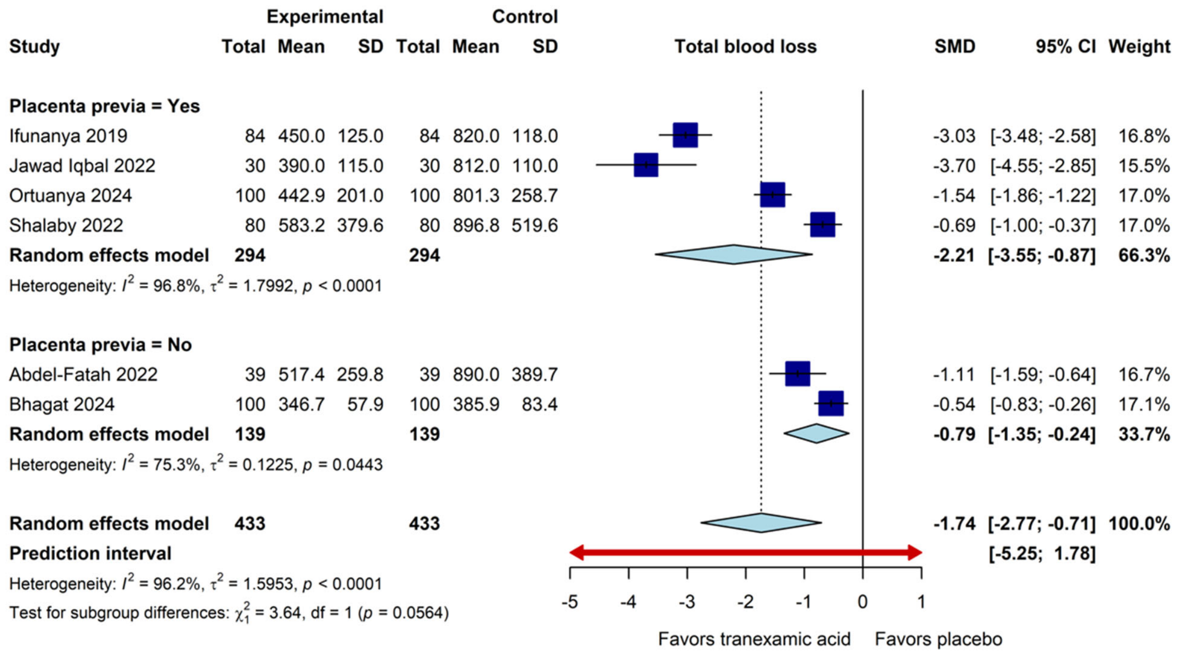

Figure S13. Subgroup analysis of total blood loss by geographic region.

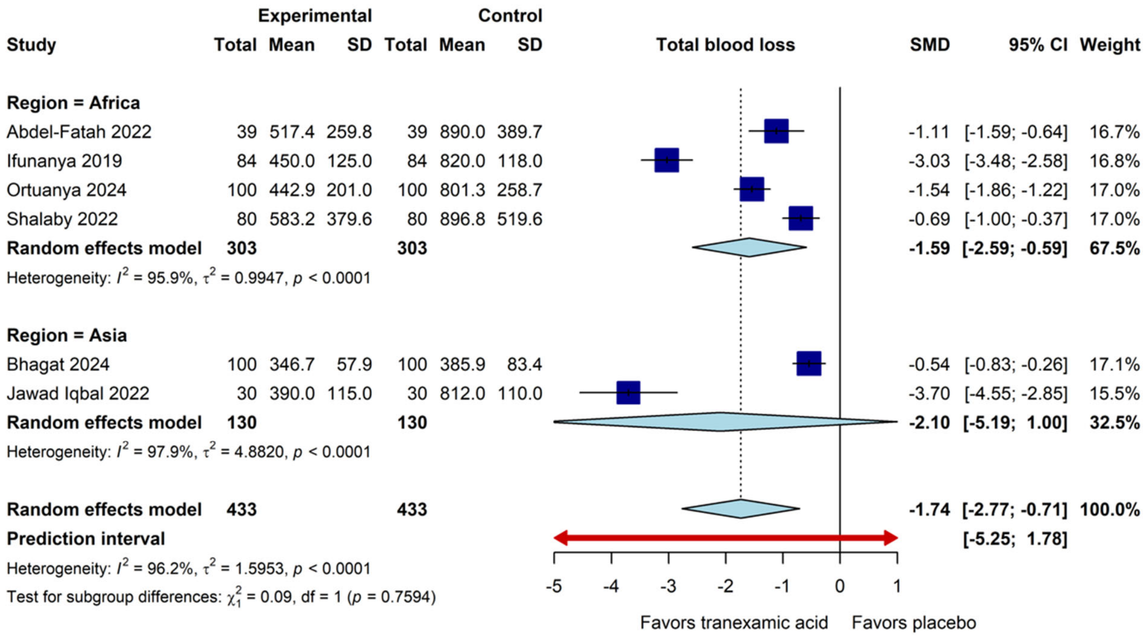

Figure S14. Subgroup analysis of total blood loss by sample size.

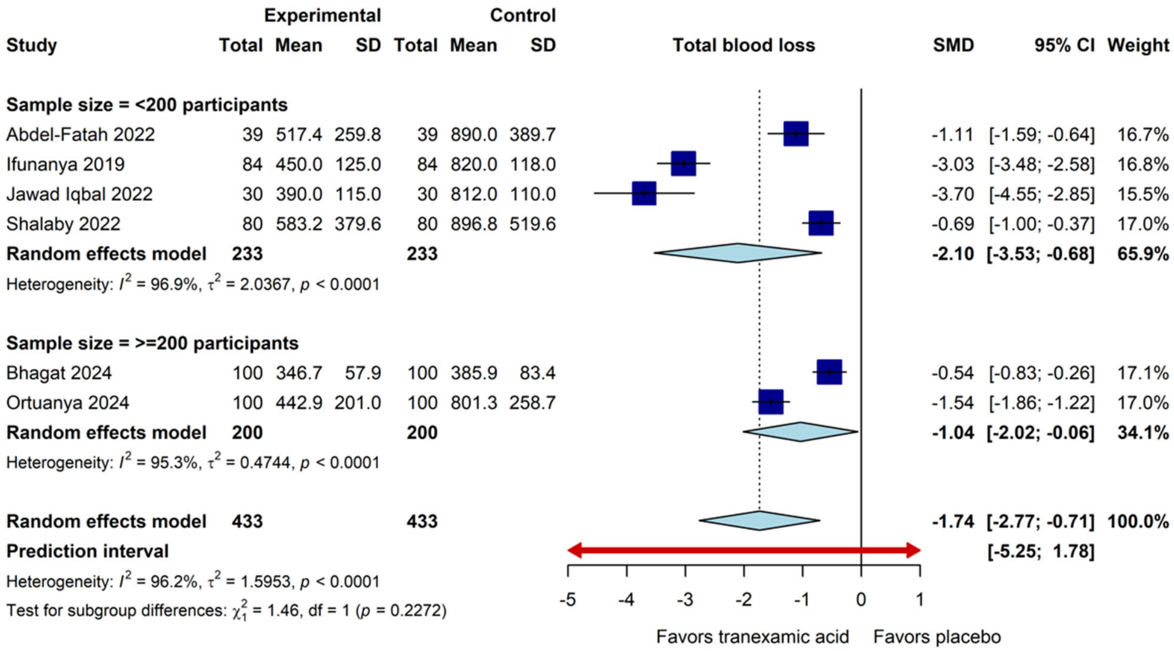

Figure S15. Subgroup analysis of total blood loss by type of cesarean delivery.

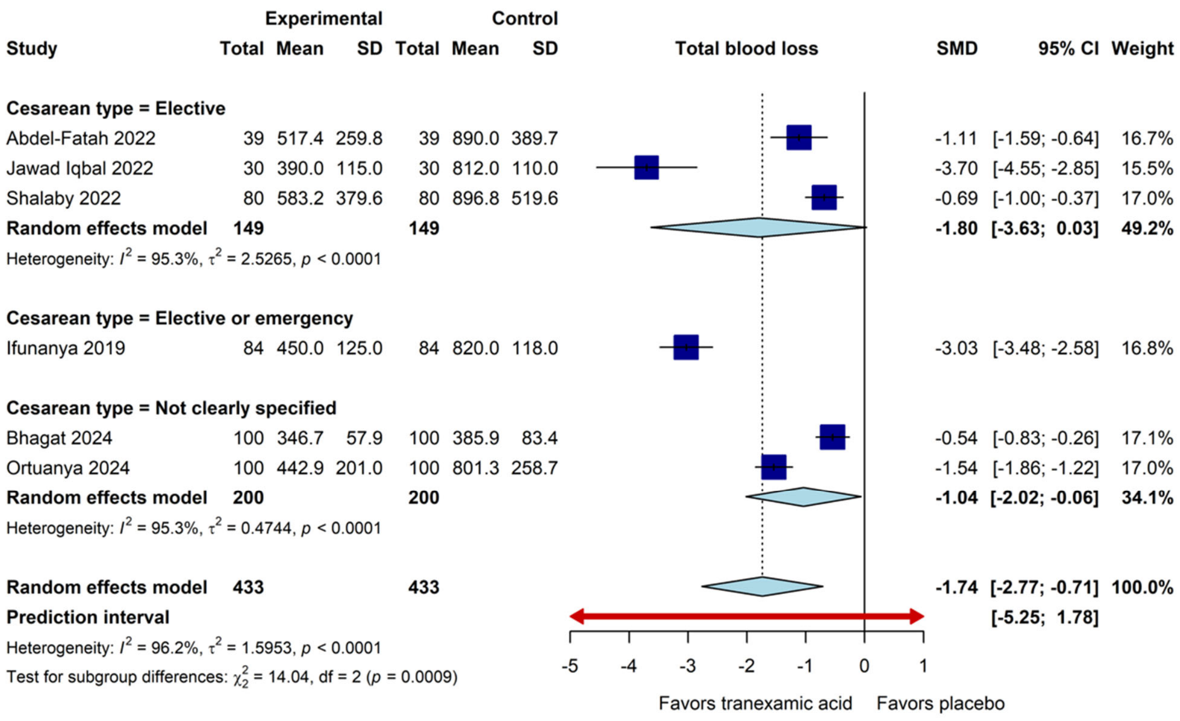

Figure S16. Subgroup analysis of total blood loss by mean maternal age.

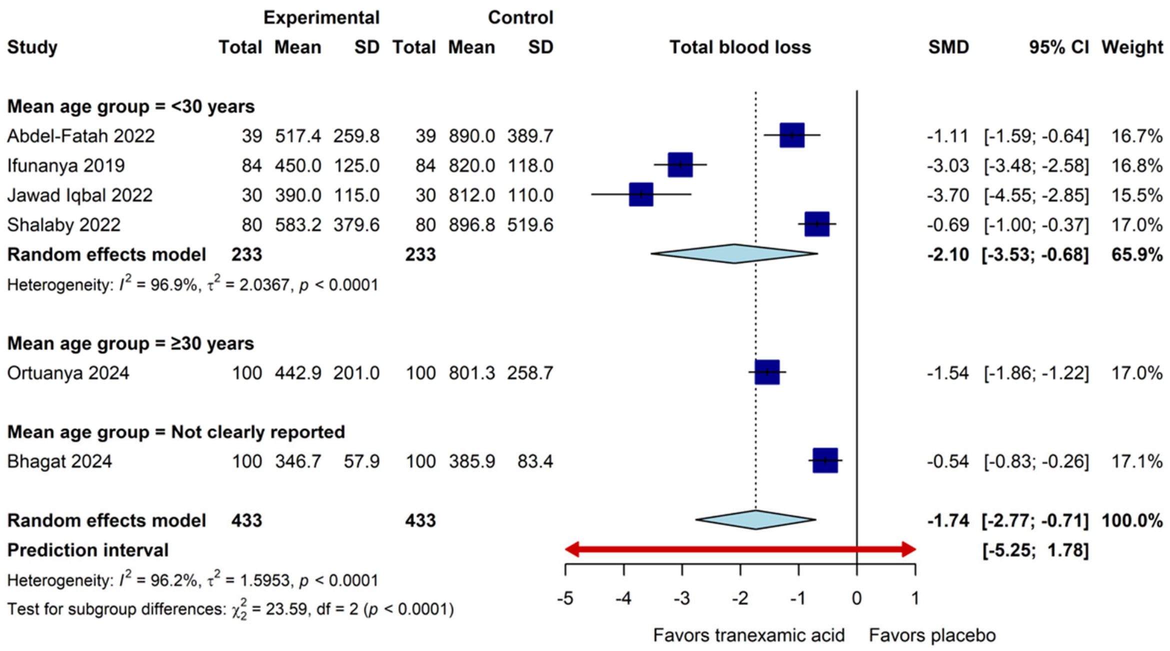

Figure S17. Meta-analysis of sensitivity analysis for intraoperative blood loss.

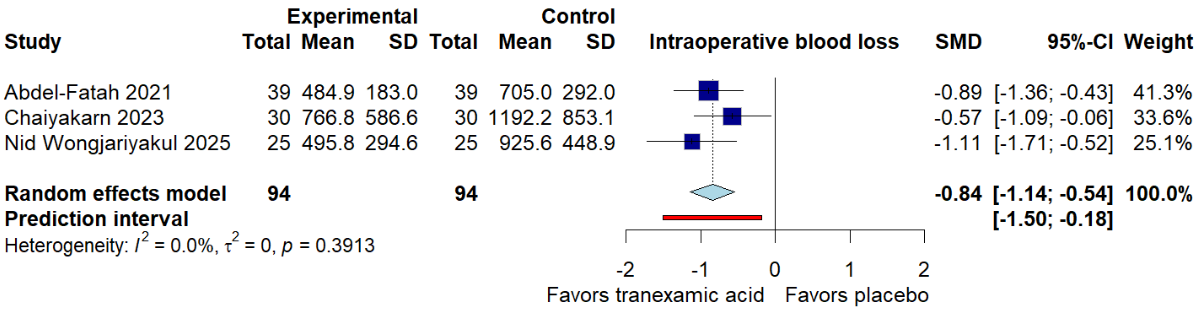

Figure S18. Meta-analysis of blood loss 2 h postpartum.

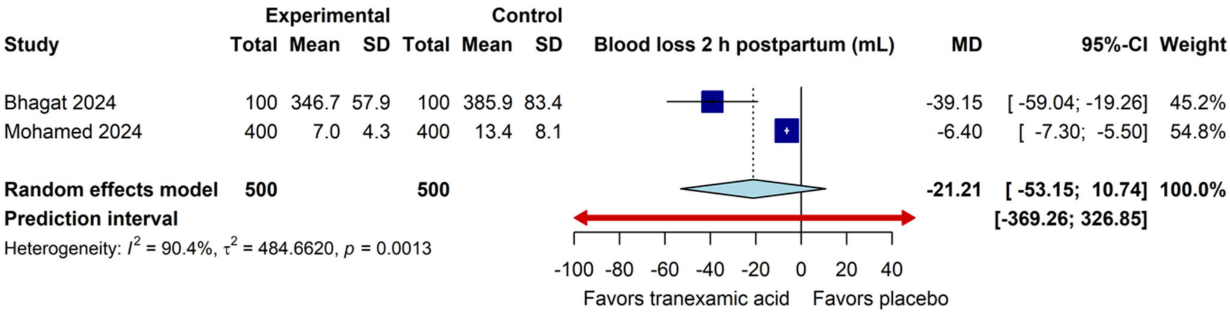

Figure S19. Baujat plot for postoperative hemoglobin using mean difference.

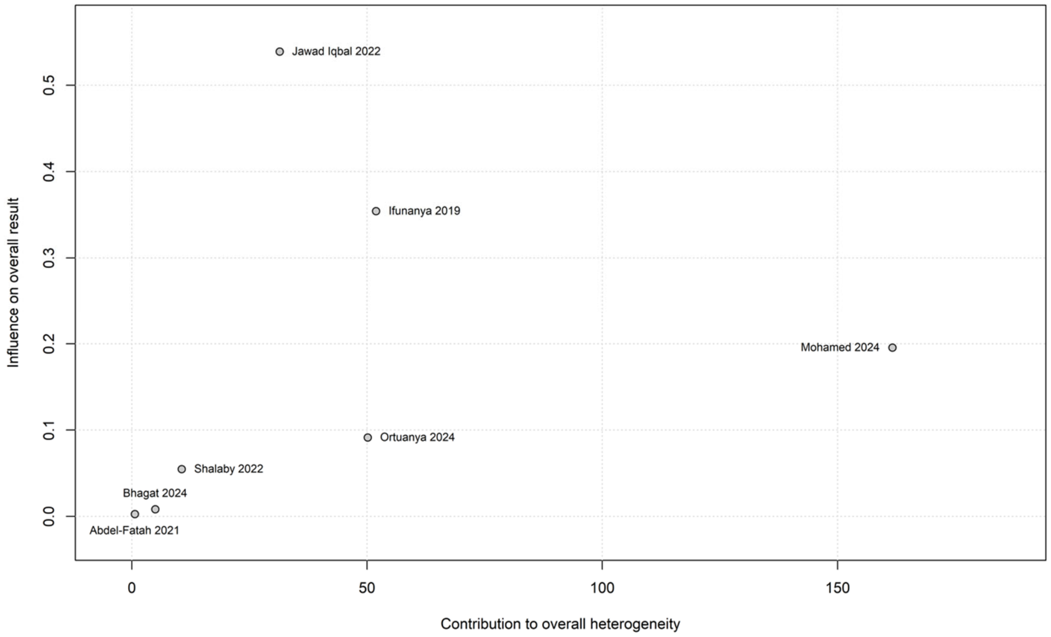

Figure S20. Leave-one-out heterogeneity analysis for postoperative hemoglobin using mean difference.

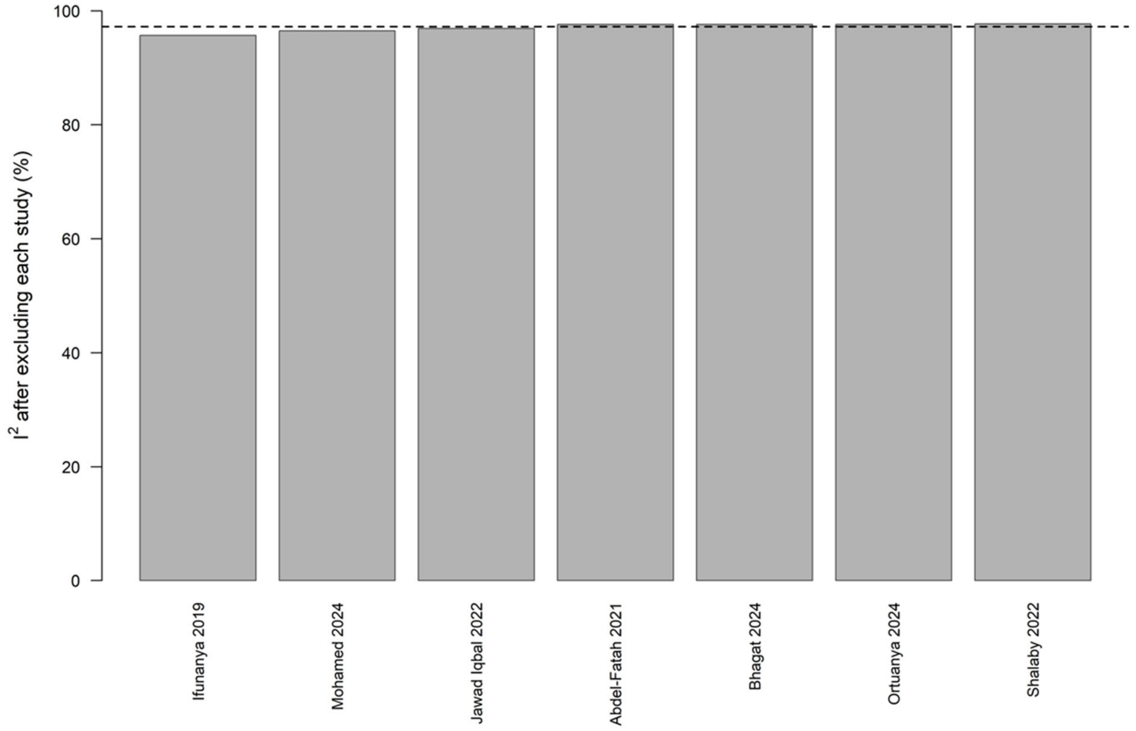

Figure S21. Meta-analysis of hospital length of stay using the mean difference.

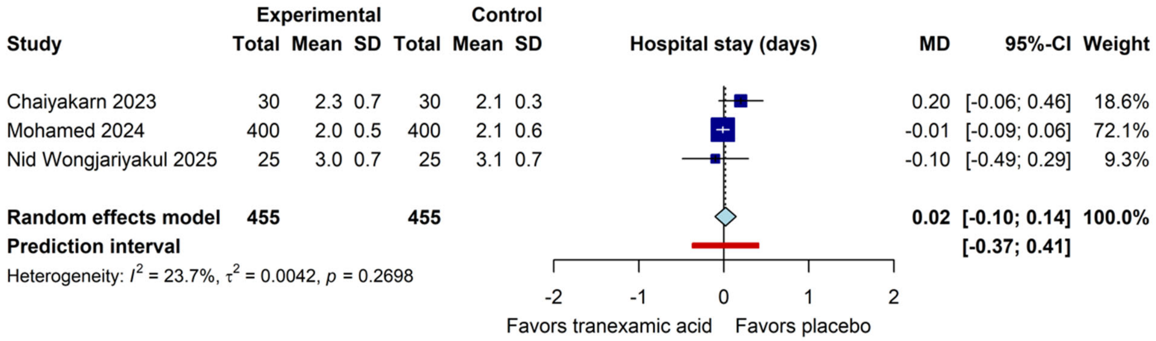

Figure S22. Meta-analysis of additional surgical intervention.

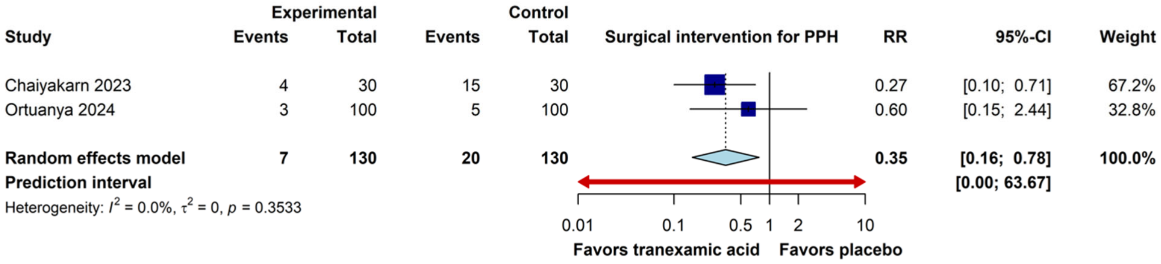

Figure S23. Meta-analysis of side effects.

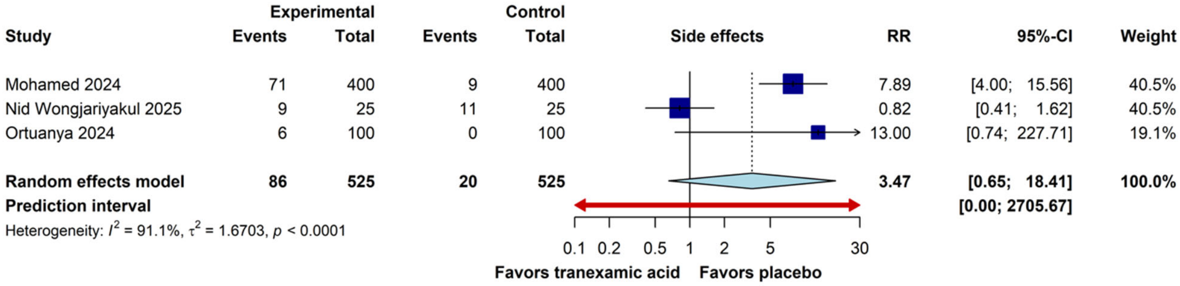

Figure S24. Meta-analysis of serious adverse events.

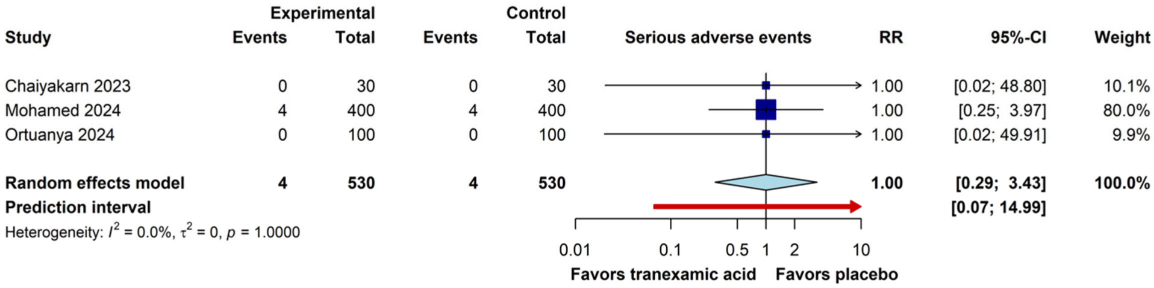

Supplement: Supplementary file 1 [file jcm-15-04630-s001.zip › jcm-4330237-supplementary.pdf]
